# Collective Writing: The Continuous Struggle for Meaning-Making

**DOI:** 10.1007/s42438-022-00320-5

**Published:** 2022-07-15

**Authors:** Petar Jandrić, Timothy W. Luke, Sean Sturm, Peter McLaren, Liz Jackson, Alison MacKenzie, Marek Tesar, Georgina Tuari Stewart, Peter Roberts, Sandra Abegglen, Tom Burns, Sandra Sinfield, Sarah Hayes, Jimmy Jaldemark, Michael A. Peters, Christine Sinclair, Andrew Gibbons

**Affiliations:** 1 Zagreb University of Applied Sciences, Croatia, and University of Wolverhampton, Wolverhampton, UK; 2 Virginia Polytechnic Institute and State University, Blacksburg, VA USA; 3 Faculty of Education and Social Work, University of Auckland, Auckland, New Zealand; 4 Chapman University, Orange, CA USA; 5 Northeast Normal University, Changchun, China; 6 The Education University of Hong Kong, Hong Kong, China; 7 Queen’s University, Belfast, UK; 8 Faculty of Education, University of Auckland, Auckland, New Zealand; 9 Auckland University of Technology, Auckland, Aotearoa New Zealand; 10 University of Canterbury, Christchurch, New Zealand; 11 University of Calgary, Calgary, Canada; 12 London Metropolitan University, London, UK; 13 University of Wolverhampton, Wolverhampton, UK; 14 Department of Education, Mid Sweden University, Sundsvall, Sweden; 15 Beijing Normal University, Beijing, China; 16 University of Edinburgh, Edinburgh, UK; 17 School of Education, Auckland University of Technology, Auckland, New Zealand

**Keywords:** Collective writing, Knowledge socialism, Educational philosophy, Postdigital, Praxis, Methodology, Openness, Academic labour, Peer co-production, Peer review, Relational epistemology, Writing as data, Indigenous knowledge, Indigenous identity, Ethics, Trust, Integrity, Collegiality, Emancipation, Positionality, Public ownership

## Abstract

This paper is a summary of philosophy, theory, and practice arising from collective writing experiments conducted between 2016 and 2022 in the community associated with the Editors’ Collective and more than 20 scholarly journals. The main body of the paper summarises the community’s insights into the many faces of collective writing. Appendix 1 presents the workflow of the article’s development. Appendix 2 lists approximately 100 collectively written scholarly articles published between 2016 and 2022. Collective writing is a continuous struggle for meaning-making, and our research insights merely represent one milestone in this struggle. Collective writing can be designed in many different ways, and our workflow merely shows one possible design that we found useful. There are many more collectively written scholarly articles than we could gather, and our reading list merely offers sources that the co-authors could think of. While our research insights and our attempts at synthesis are inevitably incomplete, ‘Collective Writing: The Continuous Struggle for Meaning-Making’ is a tiny theoretical steppingstone and a useful overview of sources for those interested in theory and practice of collective writing.

## Introduction: Herding Cats, Building Narratives (Petar Jandrić)

In 2016, Michael Peters invited me to write a 500-word contribution for the collectively authored paper ‘Toward a Philosophy of Academic Publishing’ (Peters et al. 2016). For me, collective writing was a new concept; I took a leap of faith, wrote my contribution, and eagerly waited to see what Michael would make of the article. I had so many questions. What does it mean to write together? How can we combine people’s diverse ideas and strands of thinking into a coherent whole? To explore these questions, I emulated Michael’s collective methodology and took the lead on a few collectively written articles including ‘Collective Writing: An Inquiry into Praxis’ (Jandrić et al. 2017) and ‘Postdigital Dialogue’ (Jandrić et al. 2019).

That article has indicated some possible directions for answering my questions and made it painfully obvious that no academic article, no matter how elaborate, can provide definitive answers. However, it was comforting to learn that I am not alone in asking these questions. Soon after, Michael founded the Editors’ Collective^1^ — a community of academic editors and authors interested in collective approaches to knowledge-making and dissemination. Our small community immediately began to rapidly expand, creating a torrent of collectively written articles on topics from academic publishing (Peters et al. 2016) to the arts (Peters et al. 2018).

In the intervening few years since, almost twenty mainstream academic journals have started to publish collective writings, and the community has produced so many articles that it became increasingly hard to keep track of them. Michael’s original concept has somehow become the norm; most of these articles are collections of 500-word contributions on the theme, with an introduction and conclusion by article instigators, often followed up by open review. It is probably too pretentious to say that Michael has started a new genre of academic writing, yet he did instigate a significant body of specific types of articles that have entered the mainstream extremely quickly.

In 2021, I was not at all surprised when Michael invited me to co-edit the book *The Methodology and Philosophy of Collective Writing*: *An Educational Philosophy and Theory Reader*, *Volume X* (Peters et al. 2021a). As the subtitle says, this is a collectively edited collection of the Editors’ Collective’s previously published collectively authored articles. It was only when we started to curate this book that I realised how many articles our community has published over these few short years. As we did our best to select the most relevant articles for our intended narrative, we felt almost guilty that we needed to skip so many important contributions due to obvious space limitations.

Soon after our book was published, it was collectively reviewed by Sandra Abegglen, Tom Burns, and Sandra Sinfield. Their review was extremely positive, yet they wrote a comment that indicates an important gap in our work:

Picking up the book, we expected a collection of arguments that would constitute a sort of manifesto on collective academic writing. However, opening the pages, it became clear that this is not that. Rather, it is a patchwork text, bringing together and re-presenting previously published articles of various kinds. … This leaves readers to map their own journey through the text and to draw their own conclusions on the potential of collective scholarly writing. (Abegglen, Burns, and Sinfield 2022)

Openness has so many virtues (Peters and Roberts 2011; Peters 2014; Jandrić 2018), yet it does not arrive without its own problems. In our age of viral modernity, when there is more published content than any human being can read (see Peters et al. 2020a, b, c, d; Peters et al. 2021b), we urgently need sources that are open to access and open to human understanding. This conclusion does not apply only to (scholarly) books and articles; it is just as relevant for image, film, databases, and other sources of information.

Reading our book (Peters et al. 2021a) and Abegglen, Burns, and Sinfield’s (2022) review, I identified two urgent areas for expansion of our collective work: methodology and synthesis. I emailed Michael: ‘Let’s do something about this!’ Michael agreed (his exact response is in the Conclusion), and we started working on the idea for this article.

### Methodology

To an extent, the community has experimented with the design of collective articles. For instance, *Postdigital Science and Education* has published collectively written pairs of articles (where the first article is provoking the second) (Networked Learning Editorial Collective 2021; Networked Learning Editorial Collective et al. 2021), collectively produced visual articles, where images are as important as text (Pfohl et al. 2021; Jandrić et al. 2020, 2021), collective responses to collective writings (MacKenzie et al. 2021), and so on. Yet Michael’s original design: one provocation, a patchwork of 500-word responses (with or without visuals), has stubbornly stuck with the community.

Given the standard length of academic articles, most of these articles are co-written by 20 or so authors. Some articles, such as Jandrić et al.’s Covid-19 responses (2020, 2021) may go up to 80 + authors, resulting in very long (150 + pages) reads. Most of these articles have been co-written using emails, resulting in limited opportunities for interaction between the contributors. These factors bring about certain consequences, some of which have become visible only at scale. To mention just one: reading and making sense of a collective article consisting of ten or twenty 500-word contributions is an exciting experience; reading and making sense of a 150-page article or a 350-page book consisting of 500-word contributions is very hard!

### Synthesis

Most collectively written articles have a different publishing trajectory from standard academic articles. Leading authors typically contact journal editors before the article is made, looking for expressions of interest and help in conceptualisation. More often than not, they also ask for some ‘good’ examples. Yet collective writing arrives in so many shapes and hues, and results in such different articles, that it is very hard to help those who want to instigate a new collective piece!

Supporting the development of collective articles as editor of *Postdigital Science and Education*, I feel an urgent need for a synthesis piece that could be used as a point of departure. Of course, ideas about collective writings cannot be systematised in one article. Yet, at the very least, an overview of current issues pertaining to collective writing, and a list of sources, would be very beneficial for further development of the field.

### Herding Cats, Building Narratives

In this article, we addressed the question of methodology by developing a slightly different collaborative process for collective writing. While we retained Michael’s original 500-word limit for individual contributions (old habits die hard!), we also developed a 9-week process in which all co-authors use a shared document to engage with the text, with plenty of opportunity for mutual review, criticism, under/over writing, and so on. We allowed contributors to choose their role (author or reviewer), and we involved reviewers from the ideational stage one (see Appendix 1).

We addressed the question of synthesis in a collective literature review, resulting in a long list of publications written collectively or written about collective writing. While our list will never be complete, many heads are better than one, and this resulted in a long list of titles that could save a lot of browsing to those interested in the theme (see Appendix 2).

In our contributions, we tried to cover as many different aspects of collective writing as we reasonably could. While the many faces of collective writing just cannot be all covered in any single article, our contributions do point out some of our main concerns at our postdigital historical moment.

It would be pretentious, and plainly wrong, to claim that this article is a one-stop resource for the theory and practice of collective writing. This is also a writing experiment, and one that merely takes our previous experiments one tiny step further. This time, however, we took special care to focus on the current issues in collective writing, expose our methodology in detail (Appendix 1), and offer a comprehensive reading list (Appendix 2). The struggle for collective meaning-making continues, and we hope that our small contribution will build at least a tiny steppingstone for future collective writers.

## Praxis and Methodology of Collective Writing (Timothy W. Luke)

The praxis and methodology of collective writing are, and have been, to a certain degree, embedded in ‘writing collectives’ for decades, if not centuries. Within many professions, like accounting, law, or science, the methods of presentation for bookkeeping, contractual agreements, or research reports often are always already in-drafts due to the logic of spreadsheets, legal sufficiency, or empirical persuasion. The content of financial statements, legal documents, and experimental results — due to professional norms, procedural brevity, or empirical reports — flow through conduits of convention that are stylised to the point that such writing often begins within or upon practically stylised authorial armatures. The next collective writer fills in contingent blanks, completes highly regulated expressive utterances, and adds insights in accord with those regarded as ‘insightful contributions’ adding to the writing collectives’ expectations of correct communicative sufficiency. The preliminary balances, initial briefs, or first reports intermix the thinking of networked professionals-of-practice, rapidly and methodically, practising professional praxis, even through handwritten and print documents.

In one register, continuously drilled dictates for collective writing standardise many documents intentionally around fixed presentational media or favoured formats, which are subconsciously adopted via the normalisation of default spreadsheet configurations, conventionalised formbooks, or routineised experimental scientific communication styles. The traffic built-up between authors and audiences endorses such material, operational, or rhetorical collective writing work-ups as pre-writs for the works that writing collectives generate, as much or more than the post-writs contributed by reviewers, printers, editors, or critics who all regulate the qualities and quantities of collective written work. The elements of individual voice, unique perspective, and original contribution are expected, but their presentation must be performed elegantly in concord with each aspect of these staging media, support staff, and styled standards.

In another register, new value-added, fresh insights, elegant arguments, or new contributions will be expressed, but often only to the extent they resonate inventively within the collective conventions authors must twirl effectively to trigger the recognition of real advances among the audience. Collective writing by writing collectives stabilises the bar for brilliance as well as banality behind, between or beneath these generative guardrails, which can both release or retard the liberatory potential of new writing collectives joining collective writing exchanges. How much the communicative channels add to, or possibly take away from, the communicated content are questions open to the collective writers to accept or reject, amend or forget, and improve or tolerate in the processes of writing collectively. Quite likely, the inescapable measure of hidden agendas, invisible networks, or latent prejudices with regard to content, style, and tone cannot easily be avoided. So their positive and negative elements can be retained as ur-textual anchors holding such experimental texts closer enough to float their shared, but never quite identical, communicative aspirations for writing collectively. Collective writing aims to organise diversity rather than replicate uniformity.

Fluid new thoughts circulate in fixed embedded channels, since university training approaches such professional communication as necessarily crafted for commercial, industrial, and organisational reception (Barnett and Bengtsen 2017). More occupations are generating such quasi-legislated standards of collective expression as professional correct cultural work. The growing expectations that academe have continuously improved capacities to ‘add value’ evolving in unison with standards of performance in non-academic communities being open to incorporating such ‘valued additions’ to maintain standards of effectiveness, profitability, or service evince new facets of the socialisation of knowledge (Peters 2021). Many conventions ‘make writing academic’, but these reworked practices of theory suggest that academic conventions are being pushed to display performative payoffs beyond those of rehearsing the ‘normal science’ traditional academic communication, continuity, and craft (Molinari 2022). Cultural workers of many types are teachers, and their cultural work often teaches most daringly at a distance through their activities as men and women of letters whose collective writings provide networks to form coalitions for learning how to contribute to, benefit from, or join with these many writing collectives (Freire 1997).

In such collaborative writing collectives, the normality of collective writing too often occluded by common professional methodological, ideological, or ethical assumptions about who can say/write as well as what, when, or why they are authorised to guide themselves in such communicative acts. Thinking and writing together can come naturally to these writing collectives. They often unconsciously presume they have the professional prerogative to guide not only themselves but also lead others by addressing those without their credentialed privileges and powers. In many ways, these experiments are an inventive assault on the traditional architecture of ‘learning through writing’, because they pit the collective writers’ well-trained capacities to write in the normalised fashion of their disciplines to flip the ‘infotecture’ of normalised individual writing to begin ‘learning through unwriting’ those conventions (Couples and Luke 1998). As this happens, the writing collective can face, and then by-pass, overcome, or reframe ‘the trained incapacity’ that years of cultivated craft have ingrained in their acting and thinking (Kahn 1979).

No one may be identified as ‘being in charge’, but their will to contribute to, steer forward, or express openly thoughts and feelings among themselves and for themselves as collective writers gives ‘a lead’ for other collectives to follow. Some ‘quality checking’ may go out the window, but audience reception, government censors, public acceptance, social movements, or underdeveloped misinterpretation soon will emerge in response to those who dare to collectively write perhaps about individual troubles/social problems (Luke 1999). Writing collectives are always linked to many more non-writing collectives that soon will engage in a range of ‘quality checks’, running from wild enthusiasm, utter neglect, and heated argument to mild amusement, nasty blowback, and baffled hesitation. Hence, collective writing begins testing its own pedagogy in ‘writing collectively’ by virtue of ‘relearning in unwriting individually’ beyond existing individual disciplinary norms in fresh networks of research testing new communicative infotectural forms.

## Openness to Collective Writing; Collective Writing as Openness… (Sean Sturm)

Collective writing is a ‘writing device’ (Callon 2002) that embodies a certain openness to the *outside* of academic writing and, perhaps, if we take the lead of Michel Foucault’s ‘The Thought of the Outside’ (1998), to the outside of writing itself, which, for him, is trans-subjective and transgressive.

Collective writing, as the term suggests, implies an openness to both the *collective* and, in turn, *writing*. Its openness to the collective is an openness to *the multiple*, which takes at least two forms. First, it involves an openness to dissensus, to the co-existence of multiple positions and perspectives, not only because it allows multiple authors to write together — and perhaps to express themselves in a way that stretches their usual field of research, mode of collaboration, or style — but also because it allows for a collective authorial position or perspective to be expressed that can be dissensual (diverse) rather than consensual (unified), in particular, for example, when the resulting text takes a more or less ‘patchworked’ form that allows for the ‘voices’ or ‘threads’ of the text to remain relatively distinct (see Guttorm, Hohti, and Paakkari 2015 on the problem of research collaboration as a humanist construct).

Second, it involves an openness to what Deleuze and Guattari (1987: 238) would call ‘alliances’, that is to say, symbioses or ‘becomings’ such as the kind of collective authorial perspective or position just described, which is, in fact, not just an alliance between human beings but also with technical ‘beings’ like the software and infrastructure through and with which the human beings interact (see Sturm in Peters et al. 2021b). Of course, the multiple positions and perspectives and the alliances that an openness to the multiple involves have more practical implications: it tends to foster both transdisciplinary collaboration (see Guattari 2015) and non-exclusive forms of intellectual property that move beyond ‘intellectual monopoly’ (Boldrin and Levine 2008), for example, through open access publishing.

This openness to the collective gives rise, in turn, to an openness to *writin*g, which is an openness to the *writtenness* of the resulting text (see Sturm in Peters et al. 2020a). Firstly, collectively written texts, in particular, patchworked texts, have tended to involve parataxis, or a logic of juxtaposition that is open to multiple interpretation (see Hayles 1990 for parataxis as characteristic of both postmodern literature and informatic technology). And they have often involved textures that involve an openness to textual experimentation, for example, by juxtaposing prose and poetry (see, for example, the ‘collaborative writing’ (Wyatt et al. 2011) or ‘post-qualitative inquiry’ (St. Pierre 2018 informed by the work of Deleuze and Guattari). Finally, they have often involved an openness to writing procedures, for example, the collage (Elbow 1999) or Surrealist *cadavre esquis* (‘exquisite corpse’, see Jandrić et al. 2020).

What collective writing’s openness to the collective and writing thus offers is an openness to the outside, that is to say, an ‘opening to the future’ (Deleuze 1988: 89) that enables both resistance to, and transformation of, academic or disciplinary norms and practices of writing.

## Collective Writing and Academic Labour (Peter McLaren)

One generative issue driving the imperatives of collective writing is that it should not be something forced on us by the academy. An analogy with the forced collectivisation of the Soviet Union’s agricultural sector during its 5-year plan or China’s People’s Communes during the Great Leap Forward is admittedly an ‘apples and oranges’ analogy, since this collective writing project is certainly not going to be the first step towards state ownership of our works nor will it bring about millions of deaths (unless from eye strain). It is doubtful that such world-historical tragedies like these will emerge from collective writing projects organised by a group of international transdisciplinary scholars (we are not being forced to join this collective, nor are we working under socialist distribution principles; private publishing has not been forbidden, and it is doubtful that any of us will be the victim of enforced pauperisation or grow hungry as a result of our efforts).

While we are not compliant, defenceless, and self-censoring human beings, there are good reasons for volunteering to experiment with writing collectively. One reason is that critical-dialogical engagement deepens our understanding of issues that require intense deliberation today, such as the transformation of the university into an increasingly inhuman, digitised corporate monstrosity — the result of an advanced digitalisation of today’s entire global economy and society which is utilising fourth industrial revolution technologies to develop the rules of the ‘social market economy’ (which is better than predatory capitalism but still opposed to socialist economic systems), expedite its services, penetrate new foreign markets, and add leverage to its own survival. These technologies include Artificial Intelligence (AI) and the analysis of ‘big data’ (machine learning, automation and robotics, nano- and bio-technology, quantum and cloud computing, 3D printing, virtual reality, new forms of energy storage, etc.).

We cannot ignore the larger political context in which the university is reimagining its priorities under the renewed mandates of a global surveillance state whose digital realms are being repurposed to, for instance, ‘tech wash’ racism in the service of crime prevention and national security. This digital restructuring ‘can be expected to result in a vast expansion of reduced-labour or laborless digital services, including all sorts of new telework arrangements, drone delivery, cash-free commerce, fintech (digitalised finance), tracking and other forms of surveillance, automated medical and legal services, and remote teaching involving pre-recorded instruction’ (Robinson 2020). Robinson also notes that the ‘post-pandemic global economy will now involve a more rapid and expansive application of digitalisation to every aspect of global society, including war and repression’. This will involve increased emotional manipulation, norm-setting, the weaponizing of political discourse, stigmatisation, and increased forms of cultural governance beyond the inherited repertoire of political gaslighting or what I call ‘ideological grooming’.

Each historical (and therefore economic) epoch in the development of society has its own ideal of what constitutes collective work and adopts a certain morality surrounding that work. Different economic systems have different moral codes. In the bourgeois-capitalist university system, a significant emphasis is placed on whose name comes first in the publication of a collective research article, for instance. Publishing with more than one author is not always valued as much as single authorship. Systems of capitalist development that value private property have corresponding moral codes about shared and collective work. Hence, the need for rethinking academic labour in this new era of cognitive capitalism could strengthen our communal immune system by creating a new ‘academic commons’ that is not powered by value production and the commodity form, beginning with digital writing communes as a means of making postdigital science work in the interests of the oppressed, rather than reproducing the corporate guardians of the transnational capitalist class.

Collective writing could follow some of the ideas developed by Josh Winn (2015) and Mike Neary (2020), who have seen the value in converting the university from a neoliberal corporation to a worker-cooperative with teachers and students assuming the roles of producers working collectively, as protagonistic agents furthering the development of socialism for the commons, for the public good. The co-operative values that inform the new design of the university could be those that inform collective writing projects such as, equality, equity, solidarity, and concern for community (Winn 2015). In this way, co-constructing knowledge through collective writing becomes a ‘red’ pedagogical practice where the teaching–learning dialectic is at play, an act of red love that is directed at overcoming difference and defending humanity from its own barbarism. While I am not calling for collective writing to become an historical repository for our better angels, the current state of world affairs does call us to engage in more proactive participation in public discourses. I do believe that collective writing can enable the group to control the means of knowledge production and potentially produce new forms of social knowledge through a ‘common ownership’ form that transforms the distinction between ‘public’ and ‘private’ in order to create an ‘academic commons’ designed for the good of the community.

## Collective Writing and Peer Co-Production, Peer Review, and Peer Systems of Control (Liz Jackson)

Collective writing can be educational for authors, while leading to high-quality outputs that benefit from collective insights and perspectives in the first instance, prior to formal peer review processes. This is due to an essential feature of collective writing: peer co-production. Through co-production, the individual author fades from view in light of a different, socially influenced and collaborative collective voice. In truth, this individual voice is always a kind of myth that is perpetuated by a neoliberal academic culture that favours competitive, comparative metrics and standards, where each person is ranked and graded according to their apparently singular performance. In other words, academic knowledge always is incremental when considered at a broad scale. Meanwhile, at the individual level:

As soon as we use a word, and expect it to be understood, we enter into an act of collaboration with both those who have used the word previously and those who are part of the same language community engaged in receiving that word, whether by listening or reading, in the here and now. (Jandrić et al. 2017: 88)

Through co-production, one instantly becomes accountable to co-authors. By ‘accountable’, I mean that they become positioned in a relationship of responsibility to co-authors, in contrast to more neoliberal, performative notions. This situation, and agreement to participate in collective writing, also immediately reflects back to the author an external view of their self and the relational, perspectival nature of their own knowledge, arguments, and claims to data. This kind of self-regulation of expression (to put it into a pedagogical language) encourages reflexivity and attempts at bridging divides through discourse. One is not alone in front of the computer screen, but always with others who will read their work, who become real embodied audiences to the work rather than anonymous, vaguely conceived peers.

Collective writing also has its own built-in peer review mechanism in that through the act of co-writing, one is subjected to others’ instant, ongoing, dynamic ‘criticism’. However, this is not the criticism of a reviewer who is hiding behind an anonymous identity — who can therefore criticise at will, without remorse, with little recourse from potentially upset, discouraged authors (Jackson et al. 2018). The peer reviewer who is a co-author and collaborator instead has already staked a claim to support the work and sees its value. Therefore, this reviewer has co-learning in mind first and foremost, and not only the responsibility of being a standard-bearer or arbiter in more abstract journal review processes. This peer reviewer aims to ensure meaning is understood by a broader audience: of two or more instead of one, initially. Through collaborative co-writing, each imprints their own understanding dialogically over time on others. Thus, there is an inbuilt internal review process of the ideas before a manuscript is submitted which can bolster the quality of the ideas and expression and the likelihood of a positive outcome through peer review.

The result is a kind of thought that is essentially collaborative, not additively different from just one person’s writing, but qualitatively different, in terms of content and perspective. This instant and ongoing peer review function of engaging in collaboration enhances quality of expression and thought before a paper gets to the formal peer review stage. This can, in turn, also help to support more positive early results in the usual processes of peer review, thereby leading to a kind of enhanced peer control of ideas, so that collective authors are not as susceptible to having their hard work revisioned at the whim of less invested external reviewers in order to be published and shared more broadly.

Other modes of formal peer review can also be helpful in relation to the ideas presented here. For example, single-anonymous review, where the reviewer knows the identities of authors, can facilitate reviewers’ more humane, constructive responses to authors, while reviews conducted by known reviewers for unknown authors can also lead to a more relational sense of collaborative, shared responsibility. Arguably, these are important innovations to more traditional double-anonymous approaches, which can lead to more supportive, collaborative modes of peer quality enhancement and control, echoing the main point of this section, that through knowing and relating to each other, processes of interpersonal reception, response, and feedback can be more constructive and productive than more individualistic approaches overall.

## Collective Writing as a Form of Relational Epistemology Without Foundations (Alison MacKenzie)

Traditional epistemology theorised knowledge as emerging from an idealised, autonomous rational thinker in isolation from social and political relations (Descartes, Kant, and many white, male western philosophers). The acquisition of knowledge (via the written word — theses, philosophical tracts, mediations) was conveyed to other autonomous rational beings learning in isolation from others — the lone learner, in great universities and in the school system (which is why we demand silence in class when individual learning is taking place; a pedagogical approach that stimulates learning, apparently). So influential was this line of enlightenment thinking that it is still hard to disabuse educators of the idea that to be a genius is to think, experiment, and write alone. Learning, however, is a social and collaborative practice, as is collective writing if approached in the same spirit; and some highly influential philosophers and psychologists in education understood this, Dewey and Vygotsky, for example, even while those who cite these thinkers continue to think that teaching and learning is an individualistic endeavour.

In contrast to the autonomous rationalist tradition, thinkers from social epistemology begin with the premise that knowledge production, acquisition, and dissemination are social: knowers are socially situated but have unequal access to, and participation in, knowledge practices, whether in the creation, production, dissemination, or conveying of knowledge. This is a point that social epistemologists (and other thinkers, of course) and, especially, feminists have sought vigorously to tell us (Lorde 1984; hooks 1992; Alcoff 1996, 2010; Fricker 2007). That analysis applies to academic writing.

As other writers using different ideas in this collective article have touched upon (Jackson, Stewart, and Roberts, for example) our everyday epistemic practices of conveying knowledge to others and making sense of our social experiences can be blocked by unequal power relations. These relations are shaped by a number of epistemic and ethical constructs, and mechanisms. For example, credibility judgements about *who* the speaker/writer is (her social identity); the identity power of individuals qua members of a social class, which shapes who is believed or trusted, and why (junior versus senior academic); identity-prejudicial stereotypes which result in social and individual biases in our judgements of the speaker’s/writer’s credibility; the social imagination, in terms of how persons are constructed (good/bad academics); and epistemic trustworthiness.

Academia can be plagued with what might be termed the vice of ‘insensitivity’ or being ‘cognitively and affectively numbed to the lives of others’ (Medina 2012). This vice means that, usually, epistemically advantaged people are inattentive to, unconcerned with, or disparaging of the experiences, problems, and aspirations of the disadvantaged or disfavoured. I am thinking of academics from the Marxist tradition; or research methods that seek to authentically include the voices of children (Burroughs and Tollefson 2016). The academically dominantly situated have the power to determine what is produced and published, particularly if they are editors, big names, reviewers of grant applications, etc.

Collaborative writing can be a form of epistemic resistance, a bulwark against such practices, and a process that can force us to acknowledge (again) that learning is social, collaborative, productive, and necessary. Transdisciplinarity, for example, a much-vaunted ideal in the academy, is not possible without collaboration and collaborative writing, most simply because our problems are too big, too complex, and too diverse (MacKenzie 2022) to be solved only by autonomous thinkers and lone writers writing from preferred standpoints (and see Peters et al. 2021c). As Jandrić et al. (2021: 75) suggest, ‘collaborative writing is a thing of learning-by-doing’ that can wrest academic writing from the dominant purview of the ‘knowledge economy’. Collaborative writing offers opportunities for unequal epistemic practices to be disrupted and to grant epistemic credibility to academic voices who find themselves on the margins of neoliberal forces.

## Collective Writing as Data (Marek Tesar)

Collective writing as data is an important subject that is often overlooked in the methodology and philosophy of collective writing. Collective writing is discussed as an experiment that moves away from the singular production of an academic article and opens up possibilities and opportunities for unexpected collaborations. It has become a part of a larger global ontological, intellectual, and conceptual project that has enabled us to connect diverse traditions and lines of thought.

Traditionally, data are often considered singular — owned by one author/researcher or research team. In contrast, if we are to think of collective writing as data, it opens up different, diverse and divergent conceptualisations of data. For example, there is a difference between data sets produced via collective writings and those, for instance, that are generated via systematic reviews. While systematic reviews produce answers to questions, collective writings produce new questions that we continue to ask ourselves.

It is important that we give attention to the notion of ‘data’. Collective writing lets us examine and see data as something which radically reconceptualises our philosophical and pedagogical attention to the concept of ‘data’. Collective writings are a great way that data can be organised and presented, and yet they do not lead to one expected solution or outcome. Perhaps we need to think of collective writing as a ‘data encounter’, rather than data mobilisation or data production. Collective writing presents and reflects a variety of ontological and epistemological stances, but it is not data-less; quite the opposite, data makes this scholarly practice of data production possible (see Koro-Ljungberg et al. 2017a, b; Koro-Ljungberg et al. 2019).

If collective writing is data, then it is important to explore the axiological questions this raises. Data are not ethically neutral, they serve a particular argument, policy, or ideology, and the lead author of the collective writing carries a particular power over the data that are included, excluded, invited, rejected, or in other ways shaped into master or minor narratives. These data can be seen as part of the accountability discourse, which is important to include in our deliberations (Ford 2020).

What data are in collective writing and how collective writing acts as data is not easy to conceptualise. Providing a definition or a definitive answer would disrupt and counter the work that collective writing is trying to achieve. What is clear is the idea of data as productive, both as a ‘noun’ or as a ‘verb’ in collective writing, and the substance it represents. Collective writing as data has enabled us to produce critical and important texts that have addressed key challenges and performed ideas of social justice (Biesta et al. 2021). Furthermore, we need to look into the future to conceptualise what the future of collective writing as data may bring us (Tesar et al. 2021)?

Finally, within the philosophical purposes of collective writing, it is important to discuss ideas around concepts, knowledge, and information. While debating concepts and the production of new knowledge is part of collective writing, the dissemination of information is perhaps not so much. The idea for caring for collective writing and the care for the data that collective writing do carry, represent, and perform is critical (Ailwood 2022).

## Repositories of Indigenous Knowledge and Identity (Georgina Tuari Stewart)

In 2013, the Philosophy of Education Society of Australasia (PESA) established a special interest group, the Indigenous Philosophy Group (IPG),^2^ which became a catalyst for ongoing collaborations resulting in collective Indigenous articles and editorials. In these collective writing projects, we applied our experience of participating in the writing experiments of the Editorial Collective to working with our Indigenous (Māori, Pacific and ‘others’) networks of friends and existing collaborators, as our first collective editorial explained (Stewart et al. 2014). The unity that comes from being Indigenous, even across email, comes from understanding the undiscussable, without need to discuss it: the ambivalence and discomfort that inevitably comes along with being Indigenous, and which makes most Indigenous scholars extremely vulnerable in their workplaces — in ways from which academic seniority offers little protection.

The second group publication made a more extended self-examination of the intersection between ‘indigenous’ and ‘philosophy’ (Mika et al. 2018). Next, we took the theme of Indigenous responses to ‘decolonization’ (Martin et al. 2020). After that, we published an article on Indigenous responses to ‘agnotology’ (Proctor 2008; Stewart et al. 2021). The latest article (Stewart et al. 2022) considers all forms of colonisation from Indigenous viewpoints on both sides of the Tasman Sea.

As Māori and Pacific university scholars in Aotearoa New Zealand, we are often ‘flying solo’ in our teams or departments, and this is one of the paradoxes of our work. We are asked and expected to represent whole collectivities of our peoples on all sorts of matters in the university, including extremely embedded questions concerning teaching, learning, research, and knowledge, while often concomitantly speaking alone on a committee or in a staff meeting or teaching team. An attitude of ‘one is enough’ often seems to operate in regards to Māori and Pacific academic staff in local universities.

The notion that Māori and Pacific university students need to see role models in their lectures has supported a tendency to overload emerging Māori and Pacific academics with low-level, labour-intensive teaching duties, at the expense of supporting them to develop their research programmes. In terms of research, Māori and Pacific academics are often expected to provide a cultural ‘imprimatur’ to every research group and give advice almost on a ‘walk-in’ basis, frequently well beyond what is recognised on official workload records. In terms of academic citizenship, they may be expected to serve on many committees, with little thought given as to the reasonableness of such demands.

The sense of unity of purpose that comes from collective Indigenous philosophy writing projects may be unconventional in terms of traditional Māori and Pacific cultures, but is no less valuable in the current academic milieu.

## Collective Writing as an Ethical System: Trust, Integrity, and Collegiality (Peter Roberts)

Trust and collegiality provide the ethical glue that holds academic entities together over the long term. Trust is a key educational virtue (Freire 1997; Haynes 2020), and an ethos of collegiality is vital if the ancient ideal of a community of scholars is to be upheld (Nussbaum 2010). We live in an age, however, where both trust and collegiality have been systematically undermined. A commitment to collegiality rubs against the grain of self-interested, competitive individualism, and appeals to the importance of trust — with responsibility — can fall on deaf ears in an institutional world structured by the language of accountability, performance, and measurement (Roberts 2022).

Accountability presupposes a lack of trust; it assumes that we cannot leave people to do their jobs well and must constantly monitor their activities to ensure that public money is being well spent. Accountability is based on satisfying formal procedural requirements, often within a hierarchical environment. It operates in a linear fashion (we speak of ‘lines’ of accountability) and it has an outward-facing orientation. *Being* accountable is not sufficient; we must *be seen* to be accountable. Responsibility, the partner of trust, relies more on inner conviction and is closely related to the notion of integrity. To conduct oneself with integrity is to *be* trustworthy and responsible. A spirit of collegiality both fosters the development of these qualities and is fostered by their prevalence among members of an academic group.

Collective writing can play its part in feeding the neoliberal academic machine, enhancing publication profiles, and generating further revenue through research assessment exercises. But it can also be quietly subversive, granting opportunities for scholars to be critical and creative in addressing controversial topics and themes, in the company of like-minded peers. This can be especially helpful for younger scholars who might otherwise feel reluctant to ‘stick their necks out’ because, as novices, it is not their place to do so, or for fear of being denied tenure or promotion. The work of new and emerging researchers can also sometimes not be given the attention it deserves simply because it takes time to establish credibility in a scholarly discipline or field. With collective writing efforts, a sense of shared responsibility towards others in the group can emerge. With larger collectives, the demands on a researcher’s time are also more manageable than those imposed by substantial sole-authored projects, allowing scholars to contribute more widely to educational discussion than they might initially have envisaged.

Trust is necessary on the part of those who lead collective writing projects; trust not only in the capacity of their fellow authors to deliver contributions within specified timeframes but also in the process. Collectively composing an article requires a willingness to live with, and indeed celebrate, uncertainty. Lead authors can provide a sense of direction and purpose in the guidelines they issue for contributors, but exactly what the article becomes will always be unknown. With so many diverse voices contributing to a collective writing exercise, there is a potentially liberating unpredictability in determining what will be said, by whom, in what ways. There must be trust that what emerges will be worthwhile, adding something distinctive to existing scholarly conversations, but the risks associated with this process (e.g., some possible losses in coherence and cohesiveness) must also be recognised.

For work that appears in academic journals, the integrity of the process is, from a publisher’s perspective, affirmed through peer review. But, here too, trust is paramount. Authors who accept invitations to contribute to collective articles often do so for collegial reasons — e.g., to support intellectual friends — and may be reluctant to offer their services again if the peer review process is destructive, debilitating, or mean-spirited. This is not to suggest that such experiences are common with collective writing; to the contrary, they are likely to be relatively rare, in part because many initiatives in this direction encourage a more open approach to reviewing. Equally, those who take on peer-reviewing tasks need some reassurance that their efforts in reading and responding will be appreciated and valued as an integral part of the composition process. Peer review, when undertaken promptly and constructively, is a vital form of service (to other scholars and to our fields of study) and will often demonstrably improve the quality of the work.

The face of academic communication is changing, and collective writing is likely to have a continuing presence in the new scholarly landscape. It may, however, evolve in some surprising ways, and all involved will want to keep an open mind in contemplating possibilities for the rigorous exchange of ideas in the future.

## Collective Writing as an Emancipatory Practice (Sandra Abegglen, Tom Burns, Sandra Sinfield)

Collective writing, writing produced by a group, is distinct from single authorship (see Peters et al. 2021a) — and is by its very nature transgressive (hooks 1994). It crosses boundaries, especially those of academic acceptability. It challenges and disrupts the individualistic and competitive norms of higher education (Hall 2021) and troubles the notion of the monologic construction of ideas and knowledge (Giroux and Searls Giroux 2006). This alternative, collective academic practice is akin to critical pedagogy (Freire 1970), embodying and representing the idea that education should empower and allow all participants to regain their sense of humanity, to become academics on their own terms.

For Molinari (2022) academic writing is essentially about knowledge-making; it is social and open to interpretation: in research, in teaching and in learning. It is not about closure. Murray (1972), in the vein of Molinari, calls writing a ‘process of discovery’ — a way to learn about and evaluate the world as well as a method of communication. Together, these conceptualisations of writing suggest that successful academic writing practices are more than ‘showing what you know’: they are a way of learning, better facilitated when engaged in together. We are not empty vessels, but co-producers (Carey 2013) and social constructors (Burr 2015), with rich lived lives and empowered when in dialogue (Bakhtin 1981) with knowledge-claims and with each other.

Writing for exploration and in exchange facilitates agency and creative power (Crème 2003; Abegglen et al. 2021a): a more humane academia (Abegglen et al. 2020). In this sense, academic writing becomes thinking and action (Abegglen et al. 2017): the flow of ideas for crafting, composing, reformulating. Free writing (Elbow 1998), slow writing (DeSalvo 2014; Berg and Seeber 2016), and especially, collaborative writing (Gale and Bowstead 2013) radically transform notions of what writing, knowledge, and ownership ‘is’, what it can be and how it might be challenged, developed, applied, and enacted.

Collective writing is refractive, ludic. It disrupts the performative and the normative; the undisturbed, common sense and day-to-day pattern of (academic) thinking and acting. This is the way we three write together. We open a Google Doc and free-write thoughts, ideas, observations, descriptions, opinions, and references. We write synchronously and asynchronously. We return frequently to our document — if you can ever leave this process — going over what we have contributed, finding patterns, inserting quotes, and making points. Instead of finding what we were already looking for, as Bowstead (in Gale and Bowstead 2013) says, we go where the writing takes us. We then edit — shift text around — cut and extend — and cut again. We engage in this sustained collaborative writing to produce a formal written piece that emerges from our joint playing with ideas, with our expertise and with our findings. As Elbow (1998: 28) states, ‘Producing writing, then, is not so much like filling a basin or pool once, but rather getting water to keep flowing through till finally it runs clear’.

Experiencing the power and potential of exploratory collaborative writing for ourselves, we saw even more vividly that the teaching of writing within the academy is often flawed. If addressed at all, it is as an individual problem and in decontextualised moments that remove the point and the power of writing itself (Abegglen et al. 2019). Thus, we strongly advocate for writing to be an integral part of the curriculum — more than a skill to master or the formulaic production of the perfect answer. Writing as emancipatory practice creates the hermeneutic space that enables students to come together and (also) to experience writing as a thinking process: writing to play with ideas and learn (Abegglen et al. 2021b).

As academics we need to make spaces in our classes — and in our schedules — for writing. Thinking that is shaped together — and over time. Thinking seeded perhaps by other creative activities such as collage or model making, thinking through drawing or making music. Collective writing can help students and faculty find their academic identity: we can become academic without losing ourselves but finding others in the process.

## Collective Writing as Positionality (Sarah Hayes)

Often academics encourage their research students to add a *positionality statement* to a written thesis to acknowledge their identity amid the research process they have enacted. *Positionality* attends to the social or political context that creates researcher or writer identity, including their race, class, gender, sexuality, and ability status among other influences. A *positionality statement* recognises a constitutive process whereby our individual values flow through and potentially bias our writing, but our writing in turn, further shapes our identities. Positionality may form an integral part of a research/writing process, or become a last-minute comment, such as when a supervisor suggests an examiner may ask the student more about their positionality!

Positionality raises interesting questions in the context of collective writing, about firstly, the perceived ‘ownership’ of any given writing approach. For example, what aspects of collective writing would I ‘claim’ as influenced by my own identity, or by that of the others in this article? Each of our ‘positions’ can shift as we read the ideas shared by our co-authors. Yet there are other forms of collective writing where the identity of authors is less transparent. A university policy document is often written by a group, so can it ever be ‘owned’ by an institution that commissioned it? Can it ever be free of the identity, or bias, of contributors? Yet I have never yet noticed a reflexive *positionality statement* included in a policy…

Secondly, others have commented on the role of authority and the rules that may guide, but also inhibit, writing. Positionality though is not easily argued with by any authority; it is based on personal and intimate perceptions, elements that make it powerful, hard to imitate.

Thirdly, asking how the constitutive ‘elements’ in each writer’s identity and context come to be identified is interesting too. In our postdigital society, key aspects of our identities, such as race, gender, or ability, are not isolated from data-driven Internet-based systems; they intersect with them. Each of us could be said to have a unique and fluid ‘postdigital positionality’ (Hayes 2021) as social media, algorithmic cultures, Internet of Things, or biodigital developments (Peters et al. 2021a, b, c, d) generate data that dialectically intertwines with our identities, circumstances, and locations. Torres‐Olave and Lee (2019) suggest that positionality is constructed around identities that are complex and fluid, enmeshed in power relations, and contextually bound. Thus, positionality alters for writers too, as their work (and lives) cross geographical and digital boundaries, but also temporal ones, where we each try to make sense of accelerated experiences of writing to deadlines in globalised, capitalist society across different media (Hayes and Jandrić 2017).

So, how might positionality be understood and responded to in collective writing, across time, space, and physical and virtual locations, as we write and connect ideas? Is positionality in collective writing collaborative, individual, or both? When I led a Masters’ course in Education, I asked participants to undertake a free writing task, sat together in a room. They wrote and commented on each other’s handwritten texts. When such an exercise is conducted online how does this alter individual and collective positionality, if at all? Recognising positionality in the postdigital contexts where we undertake collective writing can help to not only indicate that ‘knowledge and voice are always located in time, space, and social power’ (Barker and Jane 2016: 643) but to consider how knowledge and voice also become fragmented forms of *data*. If collective comments made by strangers on a topic in an online forum quickly become data, how does this differ when enacting a piece of collective writing? Is it a different shared ethos? What connects us, and what may divide us, as the narratives we write and comment on remain active online and gain independence from us, as the writers that first shared them? Perhaps the knowledge that our shared critically reflexive thinking and writing ‘enables people to re-write their lived experiences’ (Hayes and Jandrić 2017:16) is enough. We have co-authored a collective positionality that we have each experienced.

## Collective Writing and the Collective Public Ownership of Production and Idea-Generation: Knowledge Socialism in Terms of Different Relationships Between the University and Society (Jimmy Jaldemark)

Collective writing is not a new idea; people have thought and written together for centuries. However, the last decade’s sharp rise in the digital evolution of society in general and, more particularly, the advent of social computing through the Internet have afforded humans new possibilities to gather in groups and explicitly express their collective intelligence. With the rise of weblogs — quickly shortened to blogs — in the 1990s, the smart mobile digital devices, microblogs, and cloud-based networking in the 2000s changed the scenario for collective writing. This development, in turn, has paved the way for digital knowledge practices based on socialism.

Therefore, digital collective writing practices are forms of knowledge socialism (Peters et al. 2020a; Peters 2021). At least if writing is considered a collaborative practice — including collective ownership of the means of production — ideas are the means produced in the collective. However, in such an approach to knowledge socialism, collective writing and collaboratively produced and owned ideas link to membership in groups or organisations. Therefore, to create society-wide ownership of knowledge, groups or organisations must be linked in a larger structure to reach a societal level. From such reasoning, a conclusion is that collective writing can emerge on at least two levels: Type A on the group or organisational level and Type B on a societal level.

Building on the ideas of Barnett, collective writing as knowledge socialism embraces the relationship between the university and the society (Barnett and Bengtsen 2017; Jaldemark 2021; Peters et al. 2020a, b). Barnett discusses three modes of this relationship: the ivory tower, the factory, and the network. Analytically, these three modes are straightforward and distinct. Nevertheless, in reality, they might be mixed up and thrive in the same university.

The old-school ivory tower university does not bother to build any substantial relationship with the society. Therefore, society needs to find ways to disseminate and apply the knowledge produced. Collective writing focuses on issues that the university and its scholars think are critical, and the free mind reigns. Collective writing in terms of knowledge socialism is an internal matter for the scholars within the tower; in effect, collective writing of Type A.

In the factory, knowledge capitalism reigns, and research, teaching, and collaborative activities and initiatives start with and reflect ideas expressed in society. The knowledge produced results from the demands of public or private capital owners (Geoghegan and Pontikakis 2008). Therefore, knowledge socialism and collaborative writing in the factory mode need to focus on and relate to societal needs. Critical perspectives of knowledge are only necessary if it solves an externally expressed societal need and if it meets criteria set up by the philosophy of new public management. Ownership of the collective writing is a public affair belonging to society. Therefore, the factory mode links to Type B of collective writing.

The third mode — the networked university — is a hybrid between the university and society (Barnett and Bengtsen 2017). The networked mode emphasises a power balance between the internal knowledge barriers of the ivory tower and the demands from the capital owners in the factory; in effect, it affords another kind of knowledge socialism. Hybrid and networked knowledge practices can emerge, building on the free mind from the ivory tower and the vital link to society of the factory. This hybrid can create conditions for collective writing and collective public ownership of knowledge production to be shared between groups and organisations in society. The merging between the ivory tower and the factory can set up barriers to the thriving knowledge capitalism and new public management philosophies in the university. It could create conditions to brew knowledge socialism approaches based on merging Type A and Type B of collective writing.

## Conclusion: Collective Writing, Openness, and Co(labor)ation: Collective Research, Writing, and Pedagogy in an Era of Knowledge Socialism (Michael A. Peters)

It is always a pleasure to work with Petar. He is inventive, thorough, collaborative, intelligent, and innovative. And we have worked together over the last few years being very productive over a range of books, topics, journals, and so on. Why I start by highlighting this relationship is because it is clearly the case that we have a genuine relationship that encourages us to work together and we spark off each other. I have come to recognise it because it has happened to me more than once where a collaboration has turned into something special and different. Collective writing does not depend on this kind of relationship but it can encourage it and allow for it by putting scholars in touch with one another.

I developed these next comments in response to the helpful review of *The Methodology and Philosophy of Collective Writing* (Peters et al. 2021a) that Petar sent me (Abegglen, Burns, and Sinfield 2022). I responded immediately so the comments have a kind of immediacy.

I suggest that you and I co-edit a piece for your journal on collective writing that explores its epistemology and its experimental focus. In developing the *Educational Philosophy and Theory* (*EPAT*)^3^ uses, I focused on the natural epistemological socialism of peer review, two concepts that structured first the philosophically implicit notion of the journal as a collection (of observations, experiments, reports, etc.), then much later the peer evaluation system that controlled the quality based on the concept of (Kantian) criticism.

The journal then emerged as (an alignment of) these two ideas that were moulded into an industrial system of knowledge production. The system was based on intellectual property with the Statue of Anne that returned author rights to the creator and grew up around the legal apparatus of rights and copyright, which was quickly re-appropriated by the publishers who reduced copyright (and creation) to a minimal payment with focus on rights for use and reuse. The industrial system also controlled the form of the article mass producing a standard peer review based loosely on the methodology of science and scientific report that eclipsed the author (writing in the third person) and subjective experience to imply an eye-of-god objectivity and that allowed an easily produced mass science.

Its crucial mechanism of peer review kicked in much later to shore up the modern scientific system but was, in fact, the basis and promise of its postmodern development for the control, assessment, and ranking of quality for the industrial system. Its radical purpose was silenced and deadened and used in the purpose of maintaining industrial system quality rather than in encouraging open criticism among peers. In the digital publishing ecosystem after 1992, peer review began to emphasise aspects of peer co-production and other forms of horizontal peer development that demonstrated different forms of collegiality, co(labor)ation, and collective intelligence.

*The Methodology and Philosophy of Collective Writing* (Peters et al. 2021a) is a historical record and a ‘patchwork’ — I like this concept because of its reference to women’s work of ‘patching’ and ‘patch-work’ like much intellectual work. The Abegglen, Burns, and Sinfield (2022) review is well taken as is your suggestion that we do something together on extending the idea but collective writing as a line or argument is only one possibility that must be supplemented by the historical narrative of development, the control of academic labour, the channelling of academic writing and subjectivity, and the effacing of the implicit collective dimension of knowledge inherent in language (and recognised by Russian formalists, Wittgenstein, the Swiss and French structuralists, the French poststructuralists).

So my response to the review is to thank Sandra, Tom and Sandra for raising a legitimate criticism and to agree with you about another carefully crafted piece that examines the argument for collective writing as part of an omnibus standard for collective writing as:

1. An argument (in standard linear form).
2. An historical narrative about publishing.
3. A praxis and methodology.
4. A philosophy based on openness — open form and multiple authors — making peer reviewing central.
5. A history of the concept of peer review and peer systems of control.
6. The emergence of peer co-production.
7. A form of relational epistemology without foundations.
8. A repository of collective subjectivity.
9. An ecosystem of new (and original) ideas (without foundations).
10. An ethical system — trust, integrity, and collegiality.
11. A pedagogy.
12. Copyleft system that can also recognise and value individual contributions to the system.
13. A form of knowledge socialism based on the collective public ownership of ideas.

My first foray in an instant response: let’s do this in a carefully crafted way that should involve openness, critique, and review for the early stages to discuss co-design of the architecture, as well as its code and content.

One thing the review does not realise or recognise very well is the ways in which collective writing is but one of the methodologies of knowledge socialism which has strong conceptual and real-world overlaps to forms of peer co-production economy that includes learning, innovation, and science economies based on what I call ‘the virtues of openness’. There is more than a kindred spirit with peer review and open peer review, but also with collective research, writing, and pedagogy, especially with forms of citizen science, which is one of the forms of public knowledge cultures. Surely this has to be one of our objectives to reinforce and strengthen the understanding of these relations?

The five stages of the collective creative process (needs work):

1. Collective ideation — the emergence of novel ideas through conversation, dialogue, discussion, and sharing that does not deplete use but enhances it.
2. The sequence and architecture of ideas in an aesthetic assemblage of text production.
3. The critical review and reevaluation of text.
4. The incorporation of critics and criticism — the under/over writing.
5. The editorial process as an iterative, spiral with a pragmatic response that represents community of inquiry.

We must also emphasise:

1. ‘Arguments’ and ‘narratives’ are different but can complement each other.
2. Technically arguments operate in logical space and narratives in historical or fictional space.
3. There are different forms of argument, most inferential but hardly linear.
4. Postmodern narrative fiction disrupts the old modernist linear narratives… to begin and end anywhere and to employ nonlinear, dynamic, and interactive storylines.
5. The ‘patchwork’ is a great epistemological device because most linear narratives are ideological and unable pragmatically to respond to disruptions or breaks.

We can expand this list; maybe see it as a matrix.

We can dress up collective writing in terms of existing theory — Wittgenstein, Pierce, Bakhtin, Foucault, etc., and more contemporary sources both literary and philosophical — but, to be honest, the source for my innovation was quite pragmatic as a journal editor. I detested the neoliberal regime of performativity that created neuroses and academic anxieties taking away the joy of collaboration and writing as well as impinging on scientific purposes. As I used to say to my students (in Peters and Jandrić 2018), the journal article is ‘a dirty little industrial machine’ based on a productionist metaphysics. I wanted a concept of the paper that tried to stretch and contest the genre. We *EPAT* Editors started by offering first a clear architecture of a theme or topic that others could then develop in their own ways. I must say I was lucky with my *EPAT* and PESA colleagues at that particular time, i.e., the last five or so years.

So the experiment of collective writing in *EPAT* was developed as a mildly subversive practice, and it had other benefits: it helped to democratise publishing; it disrupted the notion of academic author; it provided relief from the tedium of the academic article; it enabled more scholars to participate in a creative process of collective writing; it provided a model suitable for quick publishing where an opinion is required almost immediately. For me, it also allowed a form of academic journalism and a form of the journal that encouraged the development of expert judgement with ‘fast reflection’. Perhaps most importantly, the *EPAT* experiment in collective writing has led to other innovations. *EPAT* now has a genre called collective writing, which has its own form of open review, and it has also been used successfully as a teaching and assessment methodology with Masters students. I have used it as such at Beijing Normal University over the last 3 years. Student feedback indicates that they enjoy collective writing and are greatly enthusiastic when it leads to a publication. I must also note that I like the idea of student-colleagues.

Collective writing, as we argued in the collection *Knowledge Socialism* (Peters et al. 2020a), is a form of collective intelligence that does not require consensus, and it is not based on the individual (ideationally, the whole is greater than the sum of its parts). The process is an emergent one, and there are synergies. It does rely on new technologies for making groups smarter, and there is an ethics of collective writing at stake, even though it has not yet been properly unpicked). Even at the small scale, collective writing is a form of self-organisation and collective adaptation at work. In this regard, there are some interesting philosophical issues to do with the group mind, with distributed cognition, coordination, and cooperation. In my view, there is much more to be understood in these terms and also in terms of devising new forms of feedback and cycles of criticism, as Petar has provided in this experiment. It is not the case as many scholars argue that, with collective intelligence, there is little to no centralised communication or control. Maybe it is useful to distinguish between architecture, code, and content. At the psychological level, it may be useful to recognise that cognition is not solitary, but is shaped by collective learning, practice, and memory.

This collective essay covers the ground nicely, and I am interested in seeing the interconnections, overlaps, and acknowledgement of both theoretical resources, but also properly acknowledge the sources for models of historical practice. My own approach, informed by Wittgenstein, Peirce, and Foucault (mostly) as a general background but also in relation to an editor’s problem that takes seriously knowledge capitalism and the political economy of academic publishing, was to develop with my colleagues a vehicle that provides a change of writing practice that yet still requires further application, development, and innovation. In particular, a group of us see collective writing and develop it explicitly as a form of knowledge socialism.

Neither I nor they want to lay claim to any originality or theoretical privilege. I am sure that liberal, feminist, and postcolonial scholars, for example, have and might develop their own distinctive collective practices (e.g. the conscious-raising group that borrowed from Freire) that create intellectual solidarity and companionship, especially as we move further away from the German idealistic philosophy that helped to craft the modern notion of the academic author and academic writing, especially with new AI publishing technologies, automatic writing and editing, and data-driven autonomous science. I am still fascinated with the ideation phase of writing — the process of having an idea — and the collective thought experiment is a useful technique for group writing that ethically shapes our collective practice.

## Review 1: Showing the Workings of Collaborative Writing (Christine Sinclair)

Too often, a journal reviewer struggles to work out what is going on in a piece of academic writing. That is certainly not the case with this collective paper: the reviewers have been in at its birth and observed its development towards maturity. Reviewers have seen in real time how ongoing commentary and peer review supported authors to clarify and extend arguments. The origins and rationale for the paper, along with its methods of production, have been made manifest in the Introduction, Conclusion, and Appendix 1. Moreover, the overarching topic of the paper is itself about what is going on in collaborative writing. This is a paper that shows its workings.

The reviewers of this paper, then, are in an unusual position in that they are not presented with a previously unseen and supposedly finished work. This is not unprecedented in academic life; the familiarity with a postgraduate student’s work can make it difficult for their supervisor to view that same work with an objective examiner’s eye, and yet sometimes they are called on to do so. The peer reviewer’s important role features strongly in the paper, and the notion of ‘co-learning with the reviewer’ captures the combination of striving for shared understanding, offering of resources, and humility that should be a necessary part of this role, especially in a collaborative endeavour. A reviewer must make a personal synthesis of the paper, offer a view on why it works or does not, and draw a conclusion about the next step (if any).

There is much woven into the fabric of this paper. The underlying method of its production shows a guiding framework that was neither coercive nor constrained — a difficult trick, as anyone who has ever been in an ‘enforced’ collaborative writing process will testify.

**Fig. 1:**
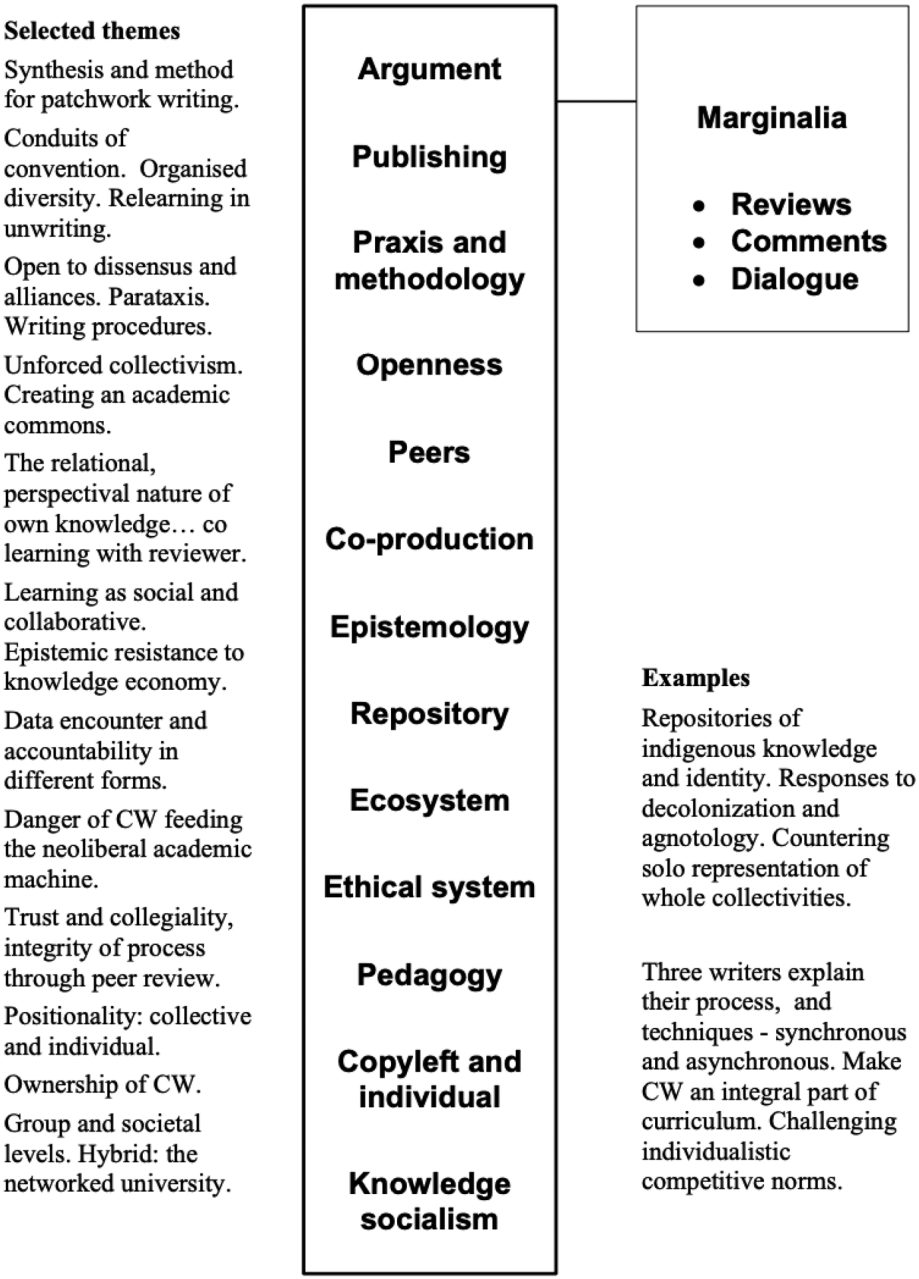
Our synthetic patchwork quilt (Christine Sinclair 2022) (CC BY NC SA 4.0)

With ‘patchwork’ writing, observation over an extended period brings concerns about how the pieces will eventually be stitched together and in which sequence. At one point, a contribution seemed to be in the wrong place, though it had great appeal with some well-crafted sentences. As I was still thinking about this, I encountered the expression ‘parataxis’ in the paper, which was new to me but exactly fitted my line of thought. Behind the scenes, some re-sequencing followed. At that point, too, the line of reasoning in the paper began to clarify for me. The paper contains many enticing words and sentences, and I captured some themes around these and mapped them to the initial list for the paper’s architecture identified in the conclusion (see Fig. 1 for the themes and examples that emerged from this process).

Many of the paper’s enticing sentences did not make it to my personal synthesis of the paper in Fig. 1. One of those supports a suitable conclusion about our openness to both the collective and its writing: we are indeed resisting and transforming traditional academic norms and practices. But I have nothing more to suggest at present. This is a valuable and well-written paper, even if we say so ourselves.

## Review 2: Good Game/Got Game/Game On/Game Over (Andrew Gibbons)

‘Collective Writing: The Continuous Struggle for Meaning-Making’ identifies a collective journey of discovery that recognises and is committed to the benefits of an openness to different approaches to writing, and methodological lessons that can be learned through and about collective writing. The methodological concern is perhaps made possible because of the experiments that led to the point of this collective work. As a reviewer and participant in some of those experiments, ‘Collective Writing: The Continuous Struggle for Meaning-Making’ has particular energy and substance in its coherence. The previous trials have created some sense of a need for a game plan. Trials and games come to mind in reviewing this collective work through a question, taking shape, about the practising of collective writing.

Thinking about practice (with connections to ludic experiences, and for writing as an integral part of the curriculum — although adding things to a curriculum can assess the fun ‘write’ out of them, more on that below) leads to thinking about the ways in which groups practise in different contexts (sports teams, bands, school of education early childhood bachelor’s programme teams, and so on). Is it possible that the kind of collective writing that builds senses of purpose and connection and trust and openness and questioning with regard to collective writing might study traditions and developments in practising?

Practice leads to performance. It is clear in this piece that the performance is focused on collective writing; however, it is also focused on decolonization and higher education, and disciplinarity and labour and peer review and …

On practising and performing peer review, this experimentation with open peer review using shared documents online and structured in such a way as to involve reviewers in the collective from the ‘stage of ideation’ is great for the study of peer review because it creates alternate experiences of peer review that then feedback into questions of what, why, and how as well as, here, where, and when. Writing a review for a piece where the authors are openly commenting and reviewing as the piece grows obstructs some reviewerly intentions, but this is not a problem — just because it has been said does not mean it cannot be said again. And, perhaps, with that dimension of a reviewer’s role taken up already, being in a sense watched by an audience as the writers work together collectively in this structured review process (I have not worked in a workshop with designers — I wonder whether there is some synergies with that design practice), the reviewer is untethered and can float elsewhere above the terrain of the piece, taking on different aspects of the horizon. And, perhaps most productively, now there is the possibility of engaging in peer review through review of the ephemeral comment functions of the authors with each other as they review themselves in the practice and performance of meaning making.

In ‘Collective Writing: The Continuous Struggle for Meaning-Making’, it is apparent that meaning-making is always a struggle and perhaps in part it is always a struggle because it is never not collective. The forgetfulness with regard that always collectiveness is perhaps the actual struggle… grappling with the collective conscious clutter that is produced by education systems and the production of particular thinking subjects who lose a sense of the we that is the I (Gibbons and Craw 2018) — a struggle to remember something some institutions may not want us to remember. Stated clearly, and with impetus for the purpose of this collective work in both practice and performance: ‘it is still hard to disabuse educators of the idea that to be a genius is to think, experiment and write alone’.

I am confident that there is an agreement that echo chamber practice fields would not offer much for collective writing without public performance. I am particularly keen to see how these collective experiments enter into different spaces of practice and performance, and the possibility of embedding curriculum experiences of collective writing. As an early childhood teacher educator, I see this work on collective writing as more than the theory and practice of collective writing; more than offering up some ways and some substance, they challenge teacher education to dive into itself and pick away at the bones of what it does.

For early childhood student teachers engaged in the study of teaching as a collective practice, collective writing contributes to recognising the ideals of collective work and ‘a certain morality surrounding that work’, and challenging the ways in which constructions of their labour and their thinking is gendered in different ways and with different implications, challenging what it means to talk about, for instance, patching and patchwork in relation to gender, and perceptions of high and low-level scholarly work that are necessary for both exploitation and emancipation as and for teachers who work in early childhood care and education.

## Appendix 1: Workflow

**Table:**

**Date**	**Stage**	**Description**	**Tasks**
**11–17 April**	Collective ideation and literature review.	Literature review and assessment of this work plan.	1. Check the list of all collectively written publications which will be published in the Appendix. 2. Add missing collectively written articles. 3. Assess this workflow; if needed, suggest changes.
**18–24 April**	The sequence and architecture of ideas.	Developing text structure and teaming up.	1. Check the provisional list of topics. Edit as you see fit – delete, merge, change sequence, add more themes. 2. Add your name next to the topic you will write about. 3. Decide whether you want to author a contribution or review the paper. If you want to serve as reviewer, write down your name in the appropriate place in the Topics. Topics and reviews are allocated on a first-come-first-served basis. If there are more people interested in a topic or a reviewer position, we strongly encourage you to team up!
**25 April – 1 May**	Writing up.	Writing up.	Write your 500-word entry.
** 2–8 May**	Critical review and re-evaluation.	Peer review.	1. Review two sections of your choice. 2. Leave your reviewer feedback as a comment attached to the section title. 3. Edit reviewed text directly (if you please).
**9–15 May**	Revising round 1	The under/over writing.	Implement reviewer comments to your section. Discuss feedback if needed.
**16–22 May Leading authors take over and produce the first draft.**			
**23–29 May**	Revising round 2	The under/over writing. Based on the first draft, reviewers write their reviews.	1. Authors: Read the whole paper and your section in particular. Finalise your section. Offer your final feedback for the paper. 2. Reviewers: Write up a 500-word review.
**30 May–5 June Leading authors take over and produce the final draft together with reviews.**			
**6–12 June**	Authorisation	The under/over writing.	Authors and reviewers: make any last changes and authorise the final version.
**13 June Paper sent to production**			

## Appendix 2: Reading List

Abegglen, S., Burns, T., & Sinfield, S. (2016). Utilising ‘critical writing exercises’ to foster critical thinking skills in first-year undergraduate students and prepare them for life outside university. *Double Helix*, *4*. 10.37514/DBH-J.2016.4.1.06.

Abegglen, S., Burns, T., & Sinfield, S. (2017). ‘Really free!’: Strategic interventions to foster students’ academic writing skills. *Journal of Educational Innovation, Partnership and Change*, *3*(1), 251–255. 10.21100/jeipc.v3i1.589.

Abegglen, S., Burns, T., & Sinfield, S. (2018). Drawing as a way of knowing: Visual practices as the route to becoming academic. *Canadian Journal for Studies in Discourse and Writing/Rédactologie*, *28*, 173–185. 10.31468/cjsdwr.600.

Abegglen, S., Burns, T., & Sinfield, S. (2021). Dialogic montage: Reflecting on playful practice in higher education. *Journal of Play in Adulthood*, *3*(2), 82–95. 10.5920/jpa.843.

Abegglen, S., Burns, T., & Sinfield, S. (2021). Editorial: Collaboration in higher education: Partnering with students, colleagues and external stakeholders. *Journal of University Teaching & Learning Practice*, *18*(7), 1–6. 10.53761/1.18.7.01.

Abegglen, S., Burns, T., & Sinfield, S. (2022). Review of Michael A. Peters, Tina Besley, Marek Tesar, Liz Jackson, Petar Jandrić, Sonja Arndt, & Sean Sturm (2021). The Methodology and Philosophy of Collective Writing: An Educational Philosophy and Theory Reader Volume X. *Postdigital Science Education*. 10.1007/s42438-022-00310-7.

Abegglen, S., Burns, T., & Sinfield, S. (2022). Supporting university staff to develop student writing: Collaborative writing as a method of inquiry. *Journal of Learning Development in Higher Education*, *23*. 10.47408/jldhe.vi23.839.

Abegglen, S., Burns, T., & Sinfield, S. (Eds.). (2021). Collaboration in higher education: Partnering with students, colleagues and external stakeholders. *Journal of University Teaching & Learning Practice*, *18*(7), Special Issue. https://ro.uow.edu.au/jutlp/vol18/iss7/. Accessed 25 May 2022.

Abegglen, S., Burns, T., & Sinfield, S. (2021). *Supporting student writing and other modes of learning and assessment: A staff guide*. Calgary: PRISM.

Abegglen, S., Burns, T., Middlebrook, D., & Sinfield, S. (2019). Disrupting academic reading: Unrolling the scroll. In L. Quinn (Ed.), *Re-imagining the curriculum: Spaces for disruption* (pp. 307–324). Stellenbosch: African Sun Media.

Ailwood, J., Lee, I-F., Arndt, S., Tesar, M., Aslanian, T., Gibbons, A., & Heimer, L. (2022). Communities of Care: A collective writing project on philosophies, politics, and pedagogies of care and education in the early years. *Policy Futures in Education*. 10.1177/14782103211064440.

Arndt, S., Asher, G., Knox, J., Ford, D. R., Hayes, S., Lăzăroiu, G., Jackson, L., Mañero Contreras, J., Buchanan, R., D’Olimpio, L., Smith, M., Suoranta, J., Pyyhtinen, O., Ryberg, T., Davidsen, J., Steketee, A., Mihăilă, R., Stewart, G., Dawson, M., Sinclair, C., & Peters, M. A. (2019). Between the blabbering noise of individuals or the silent dialogue of many: A collective response to ‘Postdigital science and education’ (Jandrić et al. 2018). *Postdigital Science and Education*, *1*(1), 446–474. 10.1007/s42438-019-00037-y.

Arndt, S., Buchanan, R., Gibbons, A., Hung, R., Madjar, A., Novak, R., Orchard, J., Peters, M. A., Sturm, S., Tesar, M., & Hood, N. (2020). Collective writing: Introspective reflections on current experience. *Educational Philosophy and Theory*. 10.1080/00131857.2020.1824782.

Bayne, S., Evans, P., Ewins, R., Knox, J., Lamb, J., Macleod, H., O’Shea, C., Ross, J., Sheail, P., & Sinclair, C. (2020). *The Manifesto for Teaching Online*. Cambridge, MA: MIT Press.

Besley, T., Jackson, L., Peters, M. A., Devine, N., Mayo, C., Stewart, G. T., White, E. J., Stengel, B., Opiniano, G. A., Sturm, S., Legg, C., Tesar, M., & Arndt, S. (2022). Philosophers and professors behaving badly: Responses to ‘named or nameless’ by Besley, Jackson, & Peters. An EPAT collective writing project. *Educational Philosophy and Theory*. 10.1080/00131857.2021.2015322.

Biesta, G., Heugh, K., Cervinkova, H., Rasiński, L., Osborne, S., Forde, D., Wrench, A., Carter, J., Säfström, C. A., Soong, H., O’Keeffe, S., Paige, K., Rigney, L.-I-, O’Toole, L., Hattam, R., Peters, M. A., & Tesar, M. (2021). Philosophy of education in a new key: publicness, social justice, and education: A South-North conversation. *Educational Philosophy and Theory*. 10.1080/00131857.2021.1929172.

Blumsztajn, A., Koopal, W., Rojahn, P., Schildermans, H., Thoilliez, B., Vlieghe, J., & Wortmann, K. (2022). Offline Memos for Online Teaching: A Collective Response to The Manifesto for Teaching Online (Bayne et al. 2020). *Postdigital Science and Education*, *4*(2), 259–270. 10.1007/s42438-022-00286-4.

Buchanan, R. A., Forster, D. J., Douglas, S., Nakar, S., Boon, H. J., Heath, T., Heyward, P., D’Olimpio, L., Ailwood, J., Eacott, S., Smith, S., Peters, M. A., & Tesar, M. (2021). Philosophy of Education in a New Key: exploring new ways of teaching and doing ethics in education in the 21st century.  *Educational Philosophy and Theory*. 10.1080/00131857.2021.1880387.

Burns, T., & Sinfield, S. (2022). *Essential study skills: The complete guide to success at university*. 5^th^ Ed. London: Sage.

Burns, T., Sinfield, S., & Abegglen, S. (2018). Case study 2: Cabinet of curiosity. *Journal of Writing in Creative Practice*, *11*(2), 211–215. 10.1386/jwcp.11.2.211_7.

Burns, T., Sinfield, S., & Abegglen, S. (2018). Case study 3: Games and board games. *Journal of Writing in Creative Practice*, *11*(2), 261–266. 10.1386/jwcp.11.2.261_7.

Burns, T., Sinfield, S., & Abegglen, S. (2018). Case study 4: Digital storytelling. *Journal of Writing in Creative Practice*, *11*(2), 275–278. 10.1386/jwcp.11.2.275_7.

Burns, T., Sinfield, S., & Abegglen, S. (2018). Case study 5: Multimodal exhibition. *Journal of Writing in Creative Practice*, *11*(2), 297–303. 10.1386/jwcp.11.2.297_7.

Burns, T., Sinfield, S., & Abegglen, S. (2018). Regenring academic writing. Case study 1: Collages. *Journal of Writing in Creative Practice*, *11*(2), 181–190. 10.1386/jwcp.11.2.181_1.

Cassidy, C., Christie, D., Coutts, N., Dunn, J., Sinclair, C., Skinner, N., & Wilson, A. (2008). Building communities of educational enquiry. *Oxford Review of Education*, *34*(2), 217–235. 10.1080/03054980701614945.

Claiborne, L. B., Cornforth, S., Crocket, K., & Manathunga, C. (2013). Exploring ethical difficulties in doctoral supervision: reflexive collaborative theorising around memory and practice. *Knowledge Cultures*, *1*(5), 39–49.

Cormier, D., Jandrić, P., Childs, M., Hall, R., White, D., Phipps, L., Truelove, I., Hayes, S., & Fawns, T. (2019). Ten Years of the Postdigital in the 52group: Reflections and Developments 2009–2019. *Postdigital Science and Education*, *1*(2), 475–506. 10.1007/s42438-019-00049-8.

Crinall, S., Rowbottom, E. C., Blom, X. P. M., & Blom, S. M. (2020). A place with no time: Re-conceptualising child–adult relations during ‘homeschooling’ in the 2020 pandemic. *Knowledge Cultures*, *8*(2), 65–81. 10.22381/KC82202010.

Czerniewicz, L., Agherdien, N., Badenhorst, J., Belluigi, D., Chambers, T., Chili, M., De Villiers, M., Felix, A., Gachago, D., Gokhale, C., Ivala, E., Kramm, N., Madiba, M., Mistri, G., Mgqwashu, E., Pallitt, N., Prinsloo, P., Solomon, K., Strydom, S., Swanepoel, M., Waghid, F., & Wissing, G. (2020). A Wake-Up Call: Equity, Inequality and Covid-19 Emergency Remote Teaching and Learning. *Postdigital Science and Education*, *2*(3), 946–967. 10.1007/s42438-020-00187-4.

Devine, N., Gresson, E., Olssen, M., Irwin, R., Coxon, E., Chueh, H., & Heraud, R. (2021). In Memoriam: Jim Marshall. *ACCESS: Contemporary Issues in Education*, *41*(1), 52-59. 10.46786/ac21.2779.

Gibbons, A., Cabral, M., & Moffett, C. (2021). Inter-galactic pedagogy, pedagogy inter-galactic: The first entries of the pedagogica intergalactica!. *Policy Futures in Education*. 10.1177/14782103211043979.

Gibbons, A., Peters, M. A., Delaune, A., Jandrić, P., Sojot, A. N., Kupferman, D. W., Tesar, M., Johansson, V., Cabral, M., Devine, N., & Hood, N.  (2021). Infantasies: An EPAT collective project. *Educational Philosophy and Theory*, *53*(14), 1442–1453. 10.1080/00131857.2020.1860749.

Gibbons, A., Peters, M. A., Stewart, G. T., Tesar, M., Boland, N., Johansson, V., de Lautour, N., Devine, N., Hood, N., & Sturm, S. (2021). *Infantologies II: Songs of the cradle, Educational Philosophy and Theory*. 10.1080/00131857.2021.1906646.

Gibbons, A., Tesar, M., Arndt, S., Kupferman, D.W., Badenhorst, D., Jackson, L., Jandrić, P., & Peters, M. A. (2020). The Highway Robber’s Road to Knowledge Socialism: A Collective Work on Collective Work. In M. A. Peters, T. Besley, P. Jandrić, & X. Zhu (Eds.) (2020). *Knowledge Socialism. The Rise of Peer Production: Collegiality, Collaboration, and Collective Intelligence* (pp. 301–325). Singapore: Springer. 10.1007/978-981-13-8126-3_15.

Hayes, S., Jopling, M., Hayes, D., Westwood, A., Tuckett, A., & Barnett, R. (2020). Raising regional academic voices (alongside data) in Higher Education (HE) debate. *Postdigital Science and Education*, *3*(2), 242–260. 10.1007/s42438-020-00131-6.

Hrastinski, S., Arkenback-Sundström, C., D. Olofsson, A., Ekström, S., Ericsson, E., Fransson, G., Jaldemark, J., Ryberg, T., Öberg, L-M., Fuentes, A., Gustafsson, U., Humble, N., Mozelius, P., Sundgren, M., & Utterberg, M. (2019). Critical Imaginaries and Reflections on Artificial Intelligence and Robots in Postdigital K-12 Education. *Postdigital Science and Education*, *1*(2), 427–445. 10.1007/s42438-019-00046-x.

Hung, R., Zhengmei, P., Kato, M., Nishihira, T., Okabe, M., Di, X., Kwak, D.-J., Hwang, K., Tschong, Y., Chien, C.-H., Peters, M. A., & Tesar, M. (2021). Philosophy of Education in a New Key: East Asia. *Educational Philosophy and Theory*, *53*(12), 1199–1214. 10.1080/00131857.2020.1772028.

Jackson, L., Alston, K., Bialystok, L., Blum, L., Burbules, N. C., Chinnery, A., Hansen, D. T., Hytten, K., Mayo, C., Norris, T., Stitzlein, S. M., Thompson, W. C., Waks, L., Peters, M. A., & Tesar, M. (2020). Philosophy of education in a New Key: Snapshot 2020 from the United States and Canada. *Educational Philosophy and Theory*. 10.1080/00131857.2020.1821189.

Jackson, L., Peters, M. A., Benade, L., Devine, N., Arndt, S., Forster, D., Gibbons, A., Grierson, E., Jandrić, P., Lazaroiu, G., Locke, K., Mihaila, R., Stewart, G., Tesar, M., Roberts, & Ozoliņs, J. (2018). Is peer review in academic publishing still working?. *Open Review of Educational Research*, *5*(1), 95–112. 10.1080/23265507.2018.1479139.

Jandrić, P., Bozkurt, A., McKee, M., Hayes, S. (2021b). Teaching in the Age of Covid-19 – A Longitudinal Study. *Postdigital Science and Education*, *3*(3), 743–770. 10.1007/s42438-021-00252-6.

Jandrić, P., Devine, N., Jackson, L., Peters, M. A., Lăzăroiu, G., Mihăilă, R., Locke, K., Heraud, R., Gibbons, A., Grierson, E., Forster, D., White, J., Stewart, G., Tesar, M., Arndt, S., Brighouse, S., & Benade, L. (2017). Collective writing: An inquiry into praxis. *Knowledge Cultures*, *5*(1), 85–109. 10.22381/KC5120177.

Jandrić, P., Hayes, D., Levinson, P., Lisberg Christensen, L., Lukoko, H. O., Kihwele, J. E., Brown, J. B., Reitz, C., Mozelius, P., Nejad, H. G., Fuentes Martinez, A., Arantes, J. A., Jackson, L., Gustafsson, U., Abegglen, S., Burns, T., Sinfield, S., Hogan, M., Kishore, P., Carr, P. R., Batarelo Kokić, I., Prinsloo, P., Grauslund, D., Steketee, A., Achieng‑Evensen, C., Komolafe, B. F., Suoranta, J., Hood, N., Tesar, M., Rose, J., Humble, N., Kirylo, J. D., Mañero, J., Monzó, L. D., Lodahl, M., Jaldemark, J., Bridges, S. M., Sharma, N., Davidsen, J., Ozoliņš, J., Bryant, P., Escaño, C., Irwin, J., Kaur, K., Pfohl, S., Stockbridge, K., Ryberg, T., Pyyhtinen, O., SooHoo, S., Hazzan, M. K., Wright, J., Hollings, S., Arndt, S., Gibbons, A., Urvashi, S., Forster, D. J., Truelove, I., Mayo, P., Rikowski, G., Stewart, P. A., Jopling, M., Stewart, G. T., Buchanan, R., Devine, N., Shukla, R., Novak, R., Mallya, M., Biličić, E., Sturm, S., Sattarzadeh, S. D., Philip, A. P., Redder, B., White, E. J., Ford, D. R., Allen, Q., Mukherjee, M., & Hayes, S. (2021). Teaching in the Age of Covid‑19 – 1 Year Later. *Postdigital Science and Education*, *3*(3), 1073–1223. 10.1007/s42438-021-00243-7.

Jandrić, P., Hayes, D., Truelove, I., Levinson, P., Mayo, P., Ryberg, T., Monzó, L.D., Allen, Q., Stewart, P.A., Carr, P.R., Jackson, L., Bridges, S., Escaño, C., Grauslund, D., Mañero, J., Lukoko, H.O., Bryant, P., Fuentes Martinez, A., Gibbons, A., Sturm, S., Rose, J., Chuma, M.M., Biličić, E., Pfohl, S., Gustafsson, U., Arantes, J.A., Ford, D.R., Kihwele, J.E., Mozelius, P., Suoranta, J., Jurjević, L., Jurčević, M., Steketee, A., Irwin, J., White, E.J., Davidsen, J., Jaldemark, J., Abegglen, S., Burns, T., Sinfield, S., Kirylo, J.D., Batarelo Kokić, I., Stewart, G.T., Rikowski, G., Lisberg Christensen, L., Arndt, S., Pyyhtinen, O., Reitz, C., Lodahl, M., Humble, N., Buchanan, R., Forster, D.J., Kishore, P., Ozoliņš, J., Sharma, N., Urvashi, S., Nejad, H.G., Hood, N., Tesar, M., Wang, Y., Wright, J., Brown, J.B., Prinsloo, P., Kaur, K., Mukherjee, M., Novak, R., Shukla, R., Hollings, S., Konnerup, U., Mallya, M., Olorundare, A., Achieng-Evensen, C., Philip, A.P., Hazzan, M.K., Stockbridge, K., Komolafe, B.F., Bolanle, O.F., Hogan, M., Redder, B., Sattarzadeh, S.D., Jopling, M., SooHoo, S., Devine, N., & Hayes, S. (2020). Teaching in The Age of Covid-19. *Postdigital Science and Education*, *2*(3), 1069–1230. 10.1007/s42438-020-00169-6.

Jandrić, P., Jaldemark. J., Hurley, Z., Bartram, B., Matthews, A., Jopling, M., Mañero, J., MacKenzie, A., Irwin, J., Rothmüller, N., Green, B., Ralston, S. J., Pyyhtinen, O., Hayes, S., Wright, J., Peters, M. A., & Tesar, M. (2021). Philosophy of education in a new key: Who remembers Greta Thunberg? Education and environment after the coronavirus. *Educational Philosophy and Theory*, *53*(14), 1421–1441. 10.1080/00131857.2020.1811678.

Jandrić, P., Ryberg, T., Knox, J., Lacković, N., Hayes, S., Suoranta, J., Smith, M., Steketee, A., Peters, M. A., McLaren, P., Ford, D. R., Asher, G., McGregor, C., Stewart, G., Williamson, B., & Gibbons, A. (2019). Postdigital Dialogue. *Postdigital Science and Education*, *1*(1), 163–189. 10.1007/s42438-018-0011-x.

Karamercan, O., Matapo, J., Kamenarac, O., Fa’avae, D. T. M., Arndt, S., Irwin, R., Kruger, F., Mika, C., Bassidou, M. Y. A., Tesar, M., & Del Monte, P. (2022). Engaging and developing community in digital spaces: Approaches from the Editorial Development Group. *Educational Philosophy and Theory*. 10.1080/00131857.2022.2041412.

Kato, M., Saito, N., Matsushita, R., Ueno, M., Izawa, S., Maruyama, Y., Sugita, H., Ono, F., Muroi, R., Miyazaki, Y., Yamana, J., Peters, M. A., & Tesar, M. (2020). Philosophy of Education in a New Key: Voices from Japan. *Educational Philosophy and Theory*. 10.1080/00131857.2020.1802819.

Koschmann, T., Hall, R. P., & Miyake, N. (Eds.). (2002). *CSCL 2; Carrying forward the conversation*. Abingdon: Routledge.

Locke, K., Gerlich, R., Godfery, M., Fraser, I., Robertson, G., & Roberts, A. (2017). The heart of the matter: A written presentation of the sixth annual Peter Fraser memorial lecture. *Knowledge Cultures*, *5*(6), 25–44. 10.22381/KC5620173.

Longley, A., Sturm, S., & Yoon, C. (2021). Kindness as water in the university. *Knowledge Cultures*, *9*(3), 184–205. 10.22381/kc93202111.

MacKenzie, A., Bacalja, A., Annamali, D., Panaretou, A., Girme, P., Cutajar, M., Abegglen, S., Evens, M., Neuhaus, F., Wilson, K., Psarikidou, K., Koole, M., Hrastinski, S., Sturm, S., Adachi, C., Schnaider, K., Bozkurt, A., Rapanta, C., Themelis, C., Thestrup, K., Gislev, T., Örtegren, A., Costello, C., Dishon, G., Hoechsmann, M., Bucio, J., Vadillo, G., Sánchez-Mendiola, M., Goetz, G., Gusso, H. L., Aldous Arantes, J., Kishore, P., Lodahl, M., Suoranta, J., Markauskaite, L., Mörtsell, S., O’Reilly, T., Reed, J., Bhatt, I., Brown, C., MacCallum, K., Ackermann, C., Alexander, C., Leah Payne, A., Bennett, R., Stone, C., Collier, A., Lohnes Watulak, S., Jandrić, P., Peters, M., & Gourlay, L. (2021). Dissolving the Dichotomies Between Online and Campus-Based Teaching: a Collective Response to The Manifesto for Teaching Online (Bayne et al. 2020) (2021). *Postdigital Science and Education*, *4*(2), 271–329. 10.1007/s42438-021-00259-z.

Martin, B., Stewart, G., Watson, B. K. i., Silva, O. K., Teisina, J., Matapo, J., & Mika, C. (2020). Situating decolonisation: An Indigenous dilemma. *Educational Philosophy and Theory*, *52*(3), 312–321. 10.1080/00131857.2019.1652164.

Mika, C., Stewart, G., Watson, K. i., Silva, K., Martin, B., Matapo, J., & Galuvao, A. (2018). What is indigenous research in philosophy of education? And what is PESA, from an indigenous perspective? *Educational Philosophy and Theory*, *50*(8), 733–739. 10.1080/00131857.2017.1317042.

Networked Learning Editorial Collective (2021). Networked Learning: Inviting Redefinition. *Postdigital Science and Education*, *3*(2), 312–325. 10.1007/s42438-020-00167-8.

Networked Learning Editorial Collective, Gourlay, L., Rodríguez-Illera, J. L., Barberà, E., Bali, M., Gachago, D., Pallitt, N., Jones, C., Bayne, S., Hansen, S. B., Hrastinski, S., Jaldemark, J., Themelis, C., Pischetola, M., Dirckinck-Holmfeld, L., Matthews, A., Gulson, K. N., Lee, K., Bligh, B., Thibaut, P.,Vermeulen, M., Nijland, F., Vrieling-Teunter, E., Scott, H., Thestrup, K., Gislev, T., Koole, M., Cutajar, M., Tickner, S., Rothmüller, N., Bozkurt, A., Fawns, T., Ross, J., Schnaider, K., Carvalho, L., Green, J. K., Hadžijusufović,M., Hayes, S., Czerniewicz, L., & Knox, J. (2021). Networked Learning in 2021: A Community Definition. *Postdigital Science and Education*, *3*(2), 326–369. 10.1007/s42438-021-00222-y.

Orchard, J., Gaydon, P., Williams, K., Bennett, P., D’Olimpio, L., Çelik, R., Shah, Q., Neusiedl, C., Suissa, J., Peters, M. A., & Tesar, M. (2021). Philosophy of education in a new key: A ‘Covid Collective’ of the Philosophy of Education Society of Great Britain (PESGB), *Educational Philosophy and Theory*, *53*(12), 1215–1228. 10.1080/00131857.2020.1838274.

Papastephanou, M., Zembylas, M., Bostad, I., Oral, S. B., Drousioti, K., Kouppanou, A., Strand, T., Wain, K., Peters, M. A., & Tesar, M. (2020). Philosophy of education in a new key: Education for justice now. *Educational Philosophy and Theory*. 10.1080/00131857.2020.1793539.

Peters, M. A. (2022). Educational Philosophies of Self-Cultivation: The Aesthetics of Collective Writing (关于修身的教育哲学：集体写作之美.Keynote address at the 2022 Annual Conference of Chinese Academy for Moral Education ( 全国德育学术委员会2021年年会), 16 April. Beijing: National Moral Education Academic Committee, School of Education of Capital Normal University, and Tian Jiabing Foundation.

Peters, M. A., Arndt, S., Tesar, M., Jackson, L., Hung, R., Mika, C., Ozolins, J. T., Teschers, C., Orchard, J., Buchanan, R., Madjar, A., Novak, R., Besley, T., Sturm, S., Roberts, P., & Gibbons. A. (2020). Philosophy of education in a new key. *Educational Philosophy and Theory*. 10.1080/00131857.2020.1759194.

Peters, M. A., Hollings, S., Zhang, M., Quainoo, E. A., Wang, H., Huang, Y., Zhou, S., Laimeche, A., Chunga, J. O., Ren, Z., Khomera, S. W., Zheng, W., Xu, R., Mou, C., & Green, B. (2021). The changing map of international student mobility. *ACCESS: Contemporary Issues in Education*, *41*(1), 7–28. 10.46786/ac21.7444.

Peters, M. A., Jandrić, P., Fuller, S., Means, A. J., Rider, S., Lăzăroiu, G., Hayes, S., Misiaszek, G. W., Tesar, M., McLaren, P., & Barnett, R. (2021). Public intellectuals in the age of viral modernity: An EPAT collective writing project. *Educational Philosophy and Theory*. 10.1080/00131857.2021.2010543.

Peters, M. A., Means, A., Neilson, D., Stewart, G. T., Jandrić, P., Sturm, S., Green, B., Ford; D. R., Fuller, S., Jackson, L., & Xue, E. (2022). ‘After Brexit and AUKUS’: A twitter-inspired collective article on changing world geopolitics and new multilateralism. *Educational Philosophy and Theory*. 10.1080/00131857.2022.2072289.

Peters, M. A., Oladele, O. M., Green, B., Samilo, A., Lv, H., Tosane, L. A., Wang, Y., Chunxiao, M., Chunga, J. O., Rulin, X., Ianina, T., Hollings, S., Barsoum Jusef, M. F., Jandrić, P., Sturm, S., Li, J., Xue, E., Jackson, L., & Tesar, M. (2020). Education in and for the Belt and Road Initiative: The Pedagogy of Collective Writing. *Educational Philosophy and Theory*, *52*(10), 1040-1063. 10.1080/00131857.2020.1718828.

Peters, M. A., Rizvi, F., McCulloch, G., Gibbs, P., Gorur, R., Hong, M., Hwang, Y., Zipin, L., Brennan, M., Robertson, S., Quay, J., Malbon, J., Taglietti, D., Barnett, R., Chengbing, W., McLaren, P., Apple, R., Papastephanou, M., Burbules, N., Jackson, L., Jalote, P., Kalantzis, M:, Cope, B., Fataar, A., Conroy, J., Misiaszek, G., Biesta, G., Jandrić, P., Choo, S., Apple, M., Stone, L., Tierney, R., Tesar, M., Besley, T., & Misiaszek, L. (2020): Reimagining the new pedagogical possibilities for universities post-Covid-19. *Educational Philosophy and Theory*. 10.1080/00131857.2020.1777655.

Peters, M. A., Tesar, M., Jackson, L., & Besley, T. (2020). *What comes after postmodernism in educational theory?* New York, NY: Routledge.

Peters, M. A., Tesar, M., Jackson, L., Besley, T., Jandrić, P., Arndt, S., & Sturm, S. (2021a). *The Methodology and Philosophy of Collective Writing: An Educational Philosophy and Theory Reader Volume X*. Abingdon and New York: Routledge.

Peters, M. A., Tesar, M., Jackson, L., Besley, T., Jandrić, P., Arndt, S., & Sturm, S. (2022). Exploring the Philosophy and Practice of Collective Writing. *Educational Philosophy and Theory*, *54*:7, 871-878.

Peters, M. A., Wang, H., Ogunniran, M. O., Huang, Y., Green, B., Chunga, J. O., Quainoo, E. A., Ren, Z., Hollings, S., Mou, C., Khomera, S. W., Zhang, M., Zhou, S., Laimeche, A., Zheng, W., Xu, R., Jackson, L., & Hayes, S. (2020). China’s Internationalised Higher Education During Covid-19: Collective Student Autoethnography. *Postdigital Science and Education*, *2*(3), 968–988. 10.1007/s42438-020-00128-1.

Peters, M. A., White, E. J., Besley, T., Locke, K., Redder, B., Novak, R., Gibbons, A., O’Neill, J., Tesar, M., & Sturm, S. (2021). Video ethics in educational research involving children: Literature review and critical discussion. *Educational Philosophy and Theory*, *53*(9), 863–880. 10.1080/00131857.2020.1717920.

Peters, M. A., White, E. J., Tesar, M., Gibbons, A., Arndt, S., Rutanen, N., Degotardi, S., Salamon, A., Browne, K., Redder, B., Charteris, J., Gould, K., Warren, A., Delaune, A., Kamenarac, O., Hood, N., & Sturm, S. (2020). Infantologies. An EPAT collective writing project. *Educational Philosophy and Theory*. 10.1080/00131857.2020.1835648.

Peters, M. A.; Jandrić, P; Irwin, R.; Locke, K.; Devine, N.; Heraud, R.; Gibbons, A.; Besley, T.; White, J.; Forster, D.; Jackson, L.; Grierson, E.; Mika, C.; Stewart, G.; Tesar, M.; Brighouse, S.; Arndt, S.; Lazariou, G.; Mihalia, R.; Bernade, L.; Legg, C.; Ozolins, J.; Roberts, P. (2016). Toward a Philosophy of Academic Publishing. *Educational Philosophy and Theory*, *48*(14), 1401-1425. 10.1080/00131857.2016.1240987.

Pfohl, S., Ayes, B., Turner, A., Amoo-Adare, E., Zecchin, M., & Borowski, M., Ito, K., Efeoglou, E., Moore, R., Wittig, M. D., Young, C., tujak, l., Thorne, A., Fletcher, B., Stevenson, D. E., Mañero, J., Maeso-Broncano, A., Mesías-Lema, J. M., Escaño, C., Hurley, Z., Spear, K., Brynjolson, N., Sanders, J. T., Lewis, T. E., & Blas, N. (2021). Simple, Dark, and Deep: Photographic Theorisations of As‑Yet Schools. *Postdigital Science and Education*, *3*(3), 793–830. 10.1007/s42438-021-00233-9.

Reader, J., Jandrić, P., Peters, M. A., Barnett, R., Garbowski, M., Lipińska, V., Rider, S., Bhatt, I., Clarke, A., Hashemi, M., Bevan, A., Trozzo, E., Mackenzie, A., Aldern, J. J., Matias, C. E., Stewart, G. T., Mika, C., McLaren, P., Fawns, T., Knox, J., Savin-Baden, M., Jackson, L., Hood, N., Tesar, M., Fuller, S., & Baker, C. (2020). Enchantment – Disenchantment – Re-Enchantment: Postdigital Relationships Between Science, Philosophy, and Religion. *Postdigital Science & Education*, *3*(3), 934–965. 10.1007/s42438-020-00133-4.

Roth, K., Mollvik, L., Alshoufani, R., Adami, R., Dineen, K., Majlesi, F., Peters, M. A., & Tesar, M. (2020). Philosophy of education in a new key: Constraints and possibilities in present times with regard to dignity. *Educational Philosophy and Theory*. 10.1080/00131857.2020.1851189.

Sardoč, M., Coady, C. A. J., Bufacchi, V., Moghaddam, F. M., Cassam, Q., Silva, D., Miščević, N., Andrejč, G., Kodelja, Z., Vezjak, B., Peters, M. A., & Tesar, M. (2021). Philosophy of education in a new key: On radicalisation and violent extremism. *Educational Philosophy and Theory*. 10.1080/00131857.2020.1861937.

Smith, L. T., Maxwell, T. K., Puke, H., & Temara, P. (2016). Indigenous knowledge, methodology and mayhem: What is the role of methodology in producing Indigenous insights? A discussion from mātauranga Māori. *Knowledge Cultures*, *4*(3), 131–156.

Stewart, G. T., Arndt, S., Besley, T., Devine, N., Forster D. J., Gibbons, A., Grierson, E., Jackson, L., Jandrić, P., Locke, K., Peters, M. A., & Tesar, M. (2017). Antipodean Theory for educational research. *Open Review of Educational Research*, *4*(1), 61-74. 10.1080/23265507.2017.1337555.

Stewart, G. T., Hogarth, M., Sturm, S., & Martin, B. (2022). Colonisation of all forms. *Educational Philosophy and Theory*. 10.1080/00131857.2022.2040482.

Stewart, G. T., MacDonald, L., Matapo, J., Fa’avae, D. T. M., Watson, B. K. i., Akiu, R. K., Martin, B., Mika, C., & Sturm, S. (2021). Surviving academic Whiteness: Perspectives from the Pacific. *Educational Philosophy and Theory*. 10.1080/00131857.2021.2010542.

Stewart, G. T., Mika, C. T. H., Cooper, G., Bidois, V., & Hoskins, T. K. (2014). Introducing the Indigenous Philosophy Group (IPG). *Educational Philosophy and Theory*, *47*(9), 851–855. 10.1080/00131857.2014.991540.

Sturm, S., Gibbons, A., & Peters, M. A. (2020). Pandemic education. *Knowledge Cultures*, *8*(3), 7–12. 10.22381/KC8320201.

Tesar, M., Duhn, I., Nordstrom, S. N., Koro, M., Sparrman, A., Orrmalm, A., Boycott-Garnett, R., MacRae, C., Hackett, A., Kuntz, A. M., Trafí-Prats, L., Boldt, G., Rautio, P., Ulmer, J. B., Taguchi, H. L., Murris, K., Kohan, W. O., Gibbons, A., Arndt, S., & Malone, K. (2021). Infantmethodologies. *Educational Philosophy and Theory*. 10.1080/00131857.2021.2009340.

Tesar, M., Guerrero, M. R., Anttila, E., Newberry, J., Hellman, A., Wall, J., Santiago-Saamong, C. R., Bodén, L., Yu, H., Nanakida, A., Diaz-Diaz, C., Xu, Y., Trnka, S., Pacini-Ketchabaw, V., Nxumalo, F., Millei, Z., Malone, K., Arndt, S. (2021). Infantographies. *Educational Philosophy and Theory*. 10.1080/00131857.2021.2009341.

Tesar, M., Hytten, K., Hoskins, T. K., Rosiek, J., Jackson, A. Y., Hand, M., Roberts, P., Opiniano, G. A., Matapo, J., St. Pierre, E. A., Azada-Palacios, R., Kuby, C. R., Jones, A., Mazzei, L. A., Maruyama, Y., O’Donnell, A., Dixon-Román, E., Chengbing, E., Huang, Z., Chen, L., Peters, M. A., & Jackson, L. (2021). Philosophy of education in a new key: Future of philosophy of education. *Educational Philosophy and Theory*. 10.1080/00131857.2021.1946792.

Tesar, M., Peters, M. A., White, E. J., Arndt, S., Charteris, J., Fricker, A., Johansson, V., Sturm, S., Hood, N., & Madjar, A. (2021). Infanticides: The unspoken side of infantologies. *Educational Philosophy and Theory*. 10.1080/00131857.2020.1854730.

Tesar, M., Peters, M. A., White, E. J., Charteris, J., Delaune, A., Thraves, G., Westbrook, F., Devine, N., Stewart, G. T. (2021). Infantilisations. *Educational Philosophy and Theory*. 10.1080/00131857.2021.1933432.

Traxler, J., Connor, S., Hayes, S., & Jandrić, P. (2021). Futures Studies, Mobilities, and the Postdigital Condition: Contention or Complement. *Postdigital Science and Education*, *4*(2), 494-518. 10.1007/s42438-021-00245-5.

Varaki, S. B., Qamsari, A. S., Sefidkhosh, M., Sajjadi, S. M., Chaboki, R. M., Kalatehjafarabadi, T. J., Saffarheidari, H., Mohammadamini, M., Karimzadeh, O., Barkhordari, R., Zarghami-Hamrah, S., Peters, M. A., & Tesar, M. (2021). Philosophy of education in a new key: Reflection on higher education in Iran. *Educational Philosophy and Theory*. 10.1080/00131857.2021.1905517.

Waghid, Y., Davids, N., Mathebula, T., Terblanche, J., Higgs, P., Shawa, L., Manthalu, C. H., Waghid, Z., Ngwenya, C., Divala, J., Waghid, F., Peters, M. A., & Tesar, M. (2020). Philosophy of education in a new key: Cultivating a living philosophy of education to overcome coloniality and violence in African Universities. *Educational Philosophy and Theory*. 10.1080/00131857.2020.1793714.

Ward, A., Christ, R. C., Kuby, C. R., & Shear, S. B. (2018). Thinking with Klosterman’s razor: Diffracting ‘reviewer 2’ and research wrongness. *Knowledge Cultures*, *6*(2), 28–50. 10.22381/KC6220183.

Yoon, C., Sturm, S., Mullen, M., Lythberg, B., Longley, A., & Harré, N. (2021). Editorial conclusion: Kindness in the review process. *Knowledge Cultures*, *9*(3), 206–219. 10.22381/kc93202112.

## Footnotes

1 See https://editorscollective.org.nz/. Accessed 12 May 2022.

2 See https://pesa.org.au/about-us/indigenous-philosophy-group. Accessed 24 May 2022.

3 See https://www.tandfonline.com/toc/rept20/current. Accessed 24 May 2022.
